# Regional variation in NAFLD prevalence and risk factors among people living with HIV in Europe: a meta-analysis

**DOI:** 10.3389/fpubh.2023.1295165

**Published:** 2024-01-04

**Authors:** Dachuan Jin, Shunqin Jin, Tao Zhou, Zhongfeng Cui, Baoqiang Guo, Guangming Li, Chunming Zhang

**Affiliations:** ^1^Clinical Laboratory, Sixth People’s Hospital of Zhengzhou, Zhengzhou, China; ^2^Department of Radiology, Hebei Medical University, Shijiazhuang, China; ^3^Department of Geriatric Medicine, Qilu Hospital of Shandong University, Jinan, China; ^4^Key Laboratory of Cardiovascular Proteomics of Shandong Province, Qilu Hospital of Shandong University, Jinan, China; ^5^Department of Life Sciences, Faculty of Science and Engineering, Manchester Metropolitan University, Manchester, United Kingdom; ^6^Department of Liver Disease, Sixth People’s Hospital of Zhengzhou, Zhengzhou, China; ^7^Department of General Surgery, Sixth People’s Hospital of Zhengzhou, Zhengzhou, China

**Keywords:** NAFLD, people living with HIV, prevalence, risk factor, Europe, meta-analysis

## Abstract

**Background and Aim:**

Europe faces an elevated risk of nonalcoholic fatty liver disease (NAFLD) among people living with HIV (PLWH), contributing to the region’s highest global burden of NAFLD. However, the prevalence of NAFLD across various European countries and regions remains unclear. This study aims to investigate the prevalence and risk factors associated with NAFLD among PLWH across European countries.

**Methods:**

A systematic search was conducted across four databases: PubMed, Embase, Web of Science, and Cochrane Library. Data on the prevalence of NAFLD, nonalcoholic steatohepatitis (NASH), and fibrosis, as well as the associated risk factors, were collected among PLWH in Europe.

**Results:**

Thirty-six studies from 13 European nations were included. The prevalence of NAFLD, NASH, and fibrosis were 42% (95%CI 37–48), 35% (95%CI 21–50) and 13% (95%CI 10–15), respectively. Male gender, BMI, waist circumference, Diabetes, hypertension, metabolic syndrome, dyslipidemia, triglycerides, HDL, LDL, ALT, AST, and years on antiretroviral therapy (ART) were found to be risk factors for NAFLD. High BMI and triglycerides were associated with NASH. Patients with high BMI and triglycerides are at increased risk of significant liver fibrosis.

**Conclusion:**

The high prevalence of NAFLD, NASH, and fibrosis among PLWH in Europe highlights the need for early screening, intervention, and increased research focus on adolescents living with HIV. Furthermore, the significant variations observed between countries and regions underscore the influence of related risk factors.

## 1 Introduction

Approximately 40 million people globally were carrying HIV, with around five thousand new cases of HIV infection occurring each day (1). However, the distribution of HIV across the world is uneven. The earliest reported cases were among men who have sex with men in Western Europe and the United States (2). But now, Eastern Europe is one of the regions with the highest HIV infection rates globally (1, 3). Additionally, with the advent of Direct-Acting Antiviral (DAA) treatment against HCV and significant improvement in the clearance rate of HBV and HCV treatment in recent years, the impact of non-infectious NAFLD on the liver has become more prominent (4, 5). On the other hand, over the past 20 years, the global coverage of antiretroviral therapy services has expanded from less than one million HIV-infected individuals in 2000 to 28.7 million in 2021 (6). With the massive expansion of HIV treatment and prevention, as well as the emergency of newer, more effective antiretroviral therapies, the global mortality rate from HIV infection has significantly decreased, greatly reducing the disparity in life expectancy with the general population (7). Under such circumstances, non-infectious chronic diseases, such as NAFLD, have a more widespread impact on the health of the PLWH population (8, 9). Recent research has found that people living with HIV (PLWH) have a higher risk of non-alcoholic fatty liver disease (NAFLD) compared to the general population (10–12), and this is believed to be related to HIV-specific factors (13).

NAFLD refers to the abnormal accumulation of triglycerides in liver cells (≥5%), with the absence of excessive alcohol consumption (men30 g/d, women20 g/d), and the exclusion of other secondary causes of fatty liver, such as genetic diseases, parenteral nutrition, viral hepatitis, etc. (14–16). NAFLD is, in fact, an umbrella term that encompasses everything from early-stage hepatic fat deposition to more severe conditions like non-alcoholic steatohepatitis (NASH), liver fibrosis, and cirrhosis (17). Since its first report in 1980, the prevalence of NAFLD has been on the rise (18). In the past two decades, NAFLD has become the most common cause of chronic liver in adults and children in developed countries (19, 20). In PLWH, chronic liver disease has also become the second leading cause of non-HIV-related mortality (21). The latest guidelines from the European AIDS Clinical Society identify PLWH as a high-risk population for NAFLD and liver fibrosis (22).

According to the recent analysis by Kalligeros et al. (23, 24), the prevalence of NAFLD in PLWH is 33.96%, significantly higher than the general population’s rate of 25.24%. On a worldwide level, Europe (42.79%) exhibits the highest prevalence of NAFLD among PLWH, greatly exceeding South America’s second-ranking rate of 35.85% (23). While Europe carries the heaviest burden of liver diseases worldwide and also has the highest NAFLD prevalence among PLWH globally, previous analyses of NAFLD prevalence in PLWH did not provide a stratified assessment of NAFLD prevalence among PLWH in different European countries and regions (25). It is widely recognized that the occurrence of HIV and NAFLD varies significantly across diverse countries, regions, populations, and ethnic groups worldwide (18, 26). However, the prevalence of NAFLD among People Living with HIV (PLWH) in varying European countries and regions remains uncertain. The primary objective of this meta-analysis is to conduct a comprehensive assessment of the prevalence and risk factors associated with NAFLD, NASH, and fibrosis in diverse European countries and regions.

## 2 Materials and methods

This systematic review and meta-analysis were conducted following the Preferred Reporting Items for Systematic Reviews and Meta-Analyses (PRISMA) guidelines and the Meta-analysis of Observational Studies in Epidemiology (MOOSE) guidelines. The protocol for this meta-analysis has been successfully registered with PROSPERO (CRD42023462228) and available in full on the http://www.crd.york.ac.uk/prospero/display.record.php?ID=CRD42023462228 (27).

### 2.1 Search strategy

We searched all relevant literature in four databases, namely PubMed, Web of Science, Embase, and Cochrane Library, from their inception up to August 20, 2023. There were no language restrictions, Table 1 provides an example of the search strategy used in PubMed.

**Table 1:** Search strategy on PubMed.

#1	"HIV"(MeSH)
#2	("HIV"[Title/Abstract] OR "human immunodeficiency virus"[Title/Abstract] OR "immunodeficiency virus human"[Title/Abstract] OR "immunodeficiency viruses human"[Title/Abstract] OR "virus human immunodeficiency"[Title/Abstract] OR "viruses human immunodeficiency"[Title/Abstract] OR "human immunodeficiency viruses"[Title/Abstract] OR "human t cell lymphotropic virus type iii"[Title/Abstract] OR "human t cell lymphotropic virus type iii"[Title/Abstract] OR "human t cell leukemia virus type iii"[Title/Abstract] OR "human t cell leukemia virus type iii"[Title/Abstract] OR "LAV-HTLV-III"[Title/Abstract] OR "lymphadenopathy associated virus"[Title/Abstract] OR "lymphadenopathy associated virus"[Title/Abstract] OR ("Lymphadenopathy-Associated"[All Fields] AND "Viruses"[Title/Abstract]) OR "virus lymphadenopathy associated"[Title/Abstract] OR "viruses lymphadenopathy associated"[Title/Abstract] OR "human t lymphotropic virus type iii"[Title/Abstract] OR "human t lymphotropic virus type iii"[Title/Abstract] OR "aids virus"[Title/Abstract] OR "aids viruses"[Title/Abstract] OR "virus aids"[Title/Abstract] OR "viruses aids"[Title/Abstract] OR "acquired immune deficiency syndrome virus"[Title/Abstract] OR "acquired immunodeficiency syndrome virus"[Title/Abstract] OR "HTLV-III"[Title/Abstract])
#3	#1 or #2
#4	"Non-alcoholic Fatty Liver Disease"(MeSH)
#5	("non-alcoholic fatty liver disease"[Title/Abstract] OR "non alcoholic fatty liver disease"[Title/Abstract] OR "NAFLD"[Title/Abstract] OR "nonalcoholic fatty liver disease"[Title/Abstract] OR "fatty liver nonalcoholic"[Title/Abstract] OR (("fatty liver"[MeSH Terms] OR ("Fatty"[All Fields] AND "Liver"[All Fields]) OR "fatty liver"[All Fields] OR ("Fatty"[All Fields] AND "Livers"[All Fields]) OR "fatty livers"[All Fields]) AND "Nonalcoholic"[Title/Abstract]) OR "liver nonalcoholic fatty"[Title/Abstract] OR (("Liver"[MeSH Terms] OR "Liver"[All Fields] OR "Livers"[All Fields] OR "liver s"[All Fields]) AND "nonalcoholic fatty"[Title/Abstract]) OR "nonalcoholic fatty liver"[Title/Abstract] OR "nonalcoholic fatty livers"[Title/Abstract] OR "nonalcoholic steatohepatitis"[Title/Abstract] OR (("fatty liver"[MeSH Terms] OR ("Fatty"[All Fields] AND "Liver"[All Fields]) OR "fatty liver"[All Fields]) AND "Nonalcoholic"[Title/Abstract]) OR "steatohepatitis nonalcoholic"[Title/Abstract])
#6	#4 OR #5
#7	#3 AND #6

### 2.2 Inclusion and exclusion criteria

Two reviewers independently screened and evaluated studies that potentially met the inclusion criteria. In case of a disagreement, a third reviewer made the final decision. The inclusion criteria were as follows: the article must report the prevalence of NAFLD and/or NASH and/or liver fibrosis in people living with HIV who are mono-infected in European countries, and it should describe the diagnostic or assessment techniques used (e.g., liver biopsy, ultrasonography, or CT scan). Exclusion criteria: studies conducted in non-European populations, studies that did not exclude excessive alcohol consumption (men≥30 g/d, women≥20 g/d), concurrent infections with HBV or HCV infections, or liver abnormalities caused by other secondary factors, animal experiments, review articles, conference abstracts, or case reports.

### 2.3 Data extraction

For each included study, we extracted the following information: author(s), publication year, nation, total number of patients (TN), study period, study design, population (adult/adolescent), diagnostic method (DxM) for NAFLD/NASH, DxM for fibrosis.

### 2.4 Quality assessment

For the assessment of study quality, we employed the JBI Critical Appraisal Checklist for Analytical Cross-Sectional Studies for both cross-sectional studies and longitudinal prospective observational studies. For the longitudinal studies, only baseline measurements were used. Each study could score a maximum of eight points. Studies with a score of five or higher were included. This assessment was independently conducted by two researchers, and any discrepancies were settled through group discussion.

### 2.5 Outcomes

The primary outcomes of interest of this study are the prevalence of NAFLD, NASH, and related liver fibrosis in European PLWH who are mono-infected, with subgroup analysis based on the patients’ countries and different regions. Additionally, the study aims to analyze the differences between adolescents and adults with HIV mono-infection in Europe. Secondary outcomes of interest include identifying potential risk factors for NAFLD, NASH, and liver fibrosis, such as age, male gender, BMI, waist circumference, hypertension, diabetes, metabolic syndrome, dyslipidemia, triglycerides, total cholesterol, high-density lipoprotein (HDL), low-density lipoprotein (LDL), alanine transaminase (ALT), aspartate transaminase (AST), CD4 count, CD4 nadir, years of HIV infection, years on antiretroviral therapy, and undetectable HIV viral load.

### 2.6 Statistical analysis

The Cochran’s Q statistic and the inconsistency index I-squared (I^2^) statistic were employed to evaluate the presence of statistical heterogeneity among studies within each model (28). In the event that the value of I^2^ surpasses 50%, we employed the constrained maximum likelihood estimator to implement the inverse variance weighted random-effects model. If the value of I^2^ did not surpass 50%, a fixed-effects model with inverse variance weighting was utilized. We utilized the Freeman-Tukey double arcsine transformation approach in order to address the issue of variance stabilization for the prevalence measure (29). In order to assess the impact of different diagnostic methods, countries, and regions on the results, we conducted subgroup analyses and meta-regression, respectively. To evaluate the presence of publication bias, we employed funnel plots and Begg’s test. Further, sensitivity analyses were performed on all studies encompassed in the investigation pertaining to NAFLD, NASH, and liver fibrosis. These analyses aimed to assess the potential influence of excluding any one study on the overall conclusions drawn from the investigation. In the meta-analysis conducted on risk factors, dichotomous variables were presented as discrete data, while continuous variables were represented as mean values accompanied by their standard deviations. This approach was employed to calculate odds ratios (OR) for dichotomous variables and mean differences for continuous variables. The 95% confidence interval (CI) was determined and provided to estimate the range within which the true prevalence in a new study is likely to fall. *p*-value was computed in order to assess their compatibility with the null hypothesis of no effect. The predetermined level of statistical significance was established at 0.05. Significance was attributed only to *p*-values that were less than or equal to 0.05. A *p*-value exceeding 0.05 was deemed to indicate a lack of statistical significance. All statistical analyses for prevalence and risk factors were conducted using STAT 15.1 software (STATA CORPORATION, College Station, TX).

## 3 Results

### 3.1 Study characteristics

**Figure 1:**
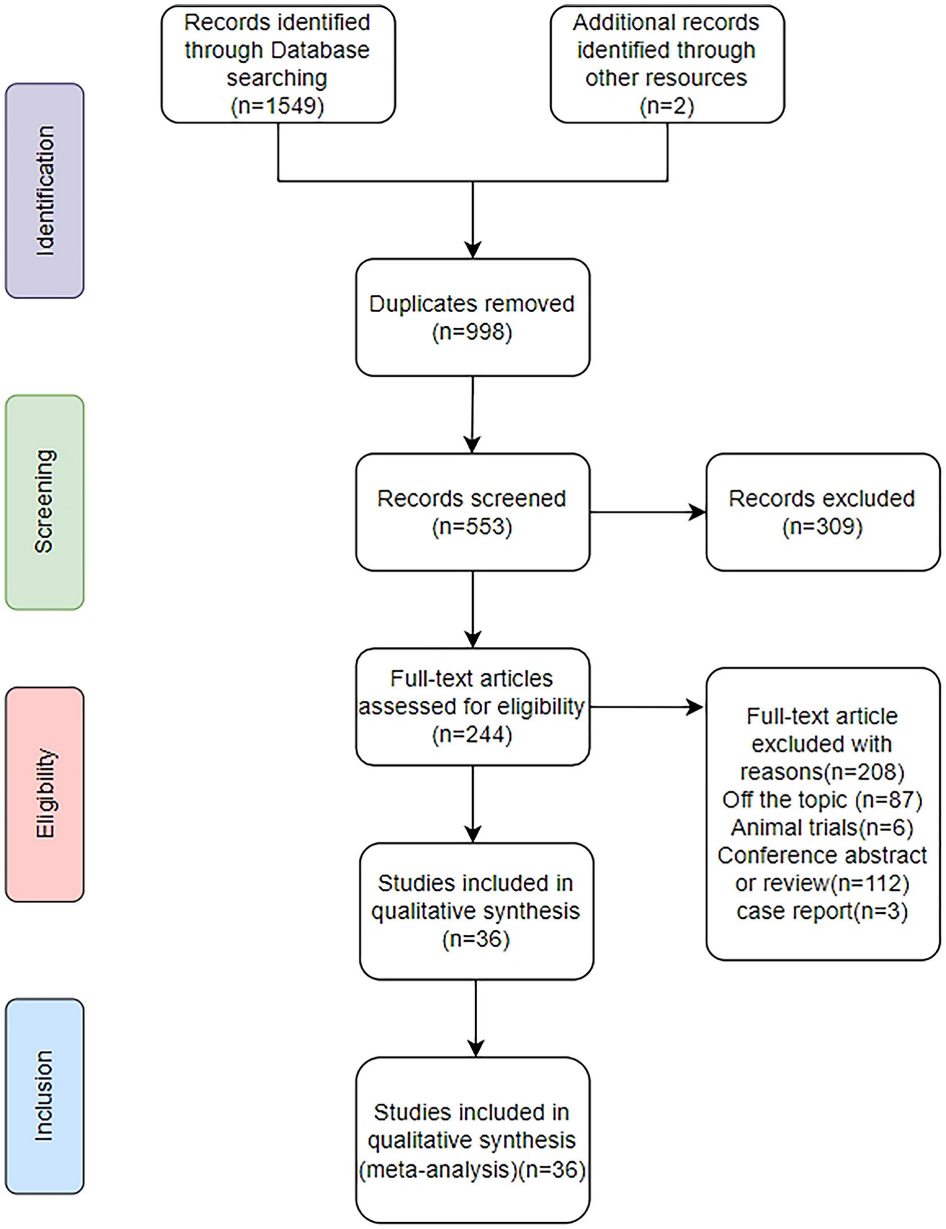
PRISMA flow diagram for included studies.

We initially retrieved a total of 1,549 articles, and after removing duplicates, we were left with 553 articles. After screening the titles and abstracts, 309 articles were excluded, leaving us with 244 articles. The excluded articles included irrelevant articles, reviews, abstracts, animal experiments, and case reports. After reviewing the full texts, a total of 36 studies met the inclusion criteria and were included in the final analysis (11, 30–64) (Figure 1). These studies were all from European countries, including the United Kingdom (42, 46), France (31, 35, 38–40, 64), Belgium (39, 40), Greece (30, 41), Italy (11, 34, 44, 48, 53, 62), Spain (33, 45, 50, 51, 55, 56), Denmark (36), Finland (37, 57), Germany (32, 39, 40, 43, 49, 58, 63), Switzerland (54), Serbia (47, 61), Austria (59), and Turkey (52, 60) (Supplementary Table S1). Most of the studies were cross-sectional in design, with a few prospective cohort studies.

### 3.2 Demographics

Only one study focused on adolescents (64), while the rest were conducted on adults. Based on the geographical distribution of the study population across different European countries, they were categorized into Central Europe (CE) (32, 43, 49, 54, 58, 59, 63), Western Europe (WE) (31, 35, 38, 42, 46, 64), Eastern Europe (EE) (47, 52, 60, 61), Southern Europe (SE) (11, 30, 33, 34, 41, 44, 45, 48, 50, 51, 53, 55, 56, 62), and Northern Europe (NE) (36, 37, 57). Two multicenter studies (39, 40) included patients from Belgium, France, and Germany. In principle, these two studies were analyzed separately and were not included in the national and regional subgroup analyses. An exception was made for the subgroup analysis of NASH prevalence, as it only included one multicenter study.

### 3.3 Study quality

**Figure 2:**
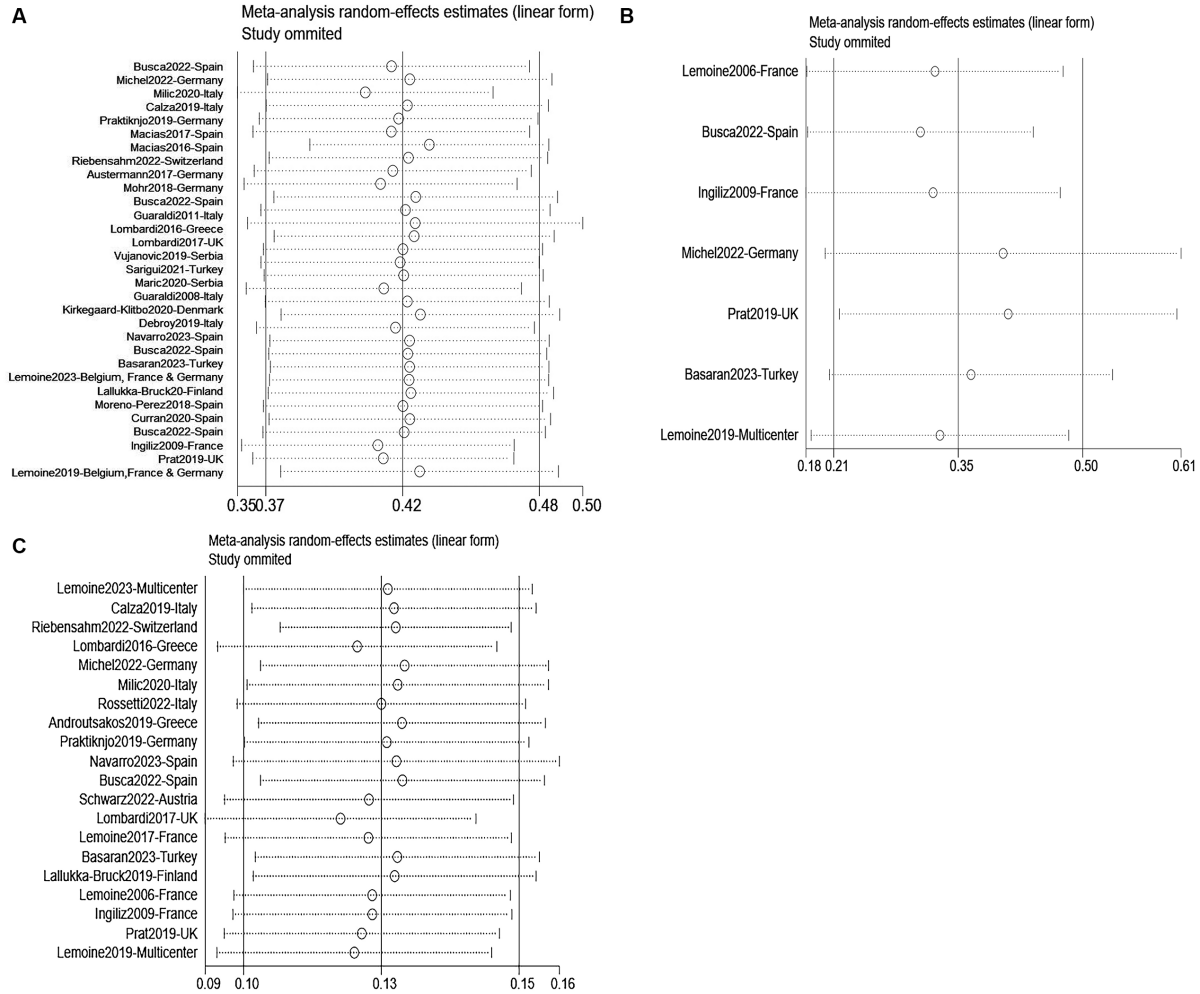
Sensitivity analysis for NAFLD **(A)**, NASH **(B)**, and liver fibrosis **(C)**.

According to the JBI Critical Appraisal Checklist, all 36 studies scored five or higher (Supplementary Table S2), and therefore, no studies were excluded due to quality issues. Except for age analysis in the risk factor studies of NAFLD, the majority of studies had Begg’s test *p*-values>0.05 (Supplementary Table S3). Thus, there was no significant publication bias. The funnel plot is roughly symmetrical on both sides, indicating no obvious publication bias (Supplementary Figures S1–S5). No literature was found through sensitivity analysis to have a significant influence on the analysis’s overall findings (Figure 2). Therefore, the results can be considered robust and reliable.

### 3.4 Prevalence of NAFLD

**Figure 3:**
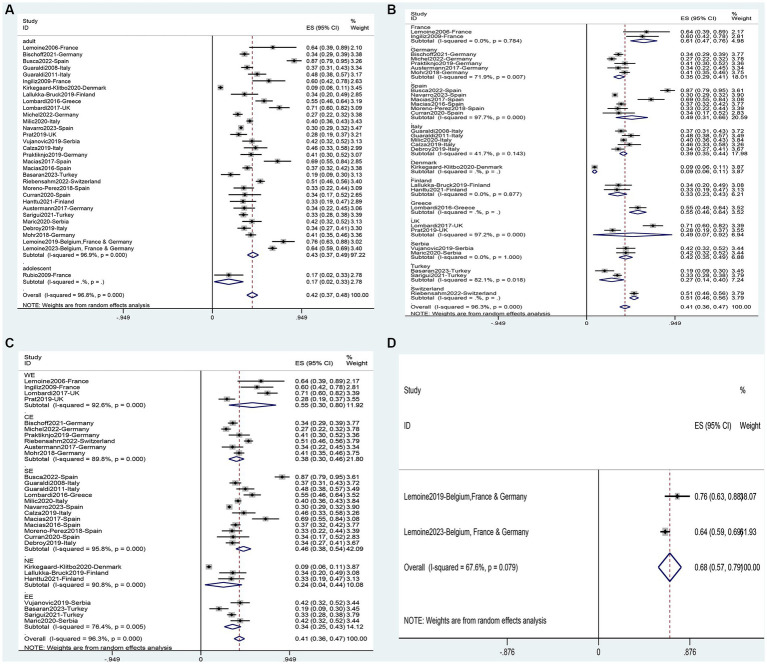
Forest plot for the prevalence of NAFLD in PLWH in Europe **(A)**, and subgrouped by nation **(B)**, region **(C)**, multicenter **(D)**.

**Figure 4:**
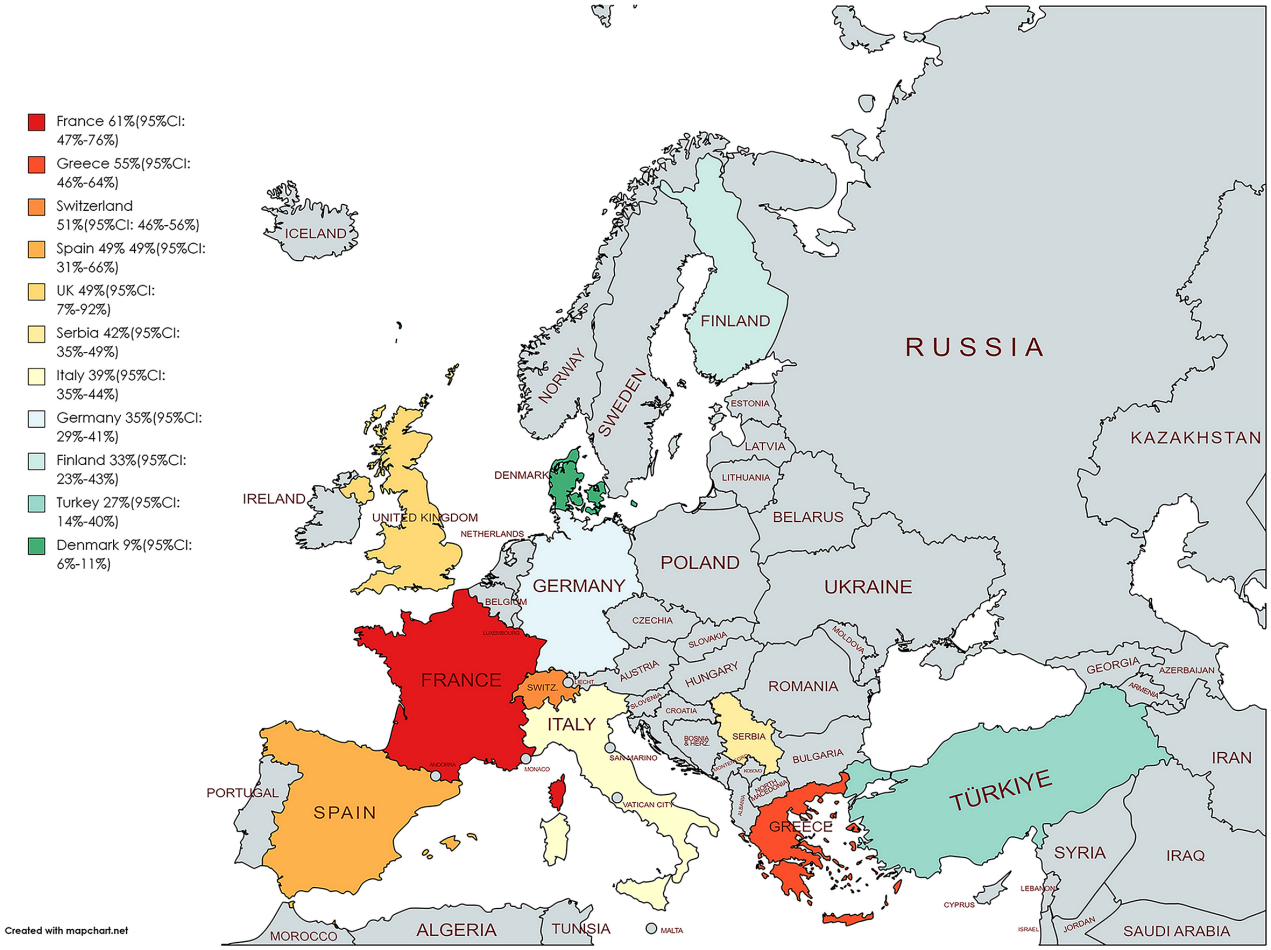
Prevalence of NAFLD in PLWH in different European countries.

A total of 32 studies were analyzed, including 1 study (64) on adolescents and 31 studies (11, 31–37, 39–52, 54–58, 60–63) on adults. It involved 9,962 adults and 23 adolescents. The analysis showed that the overall prevalence of NAFLD in the people living with HIV mono-infection in Europe was 42% (95%CI, 37–48) (Figure 3A). Subgroup analysis for different populations showed that the incidence of NAFLD in adults (43, 95%CI: 37–49) was significantly higher than in adolescents (17, 95%CI: 2–33) (Supplementary Table S4). Subgroup analysis based on national levels (Figures 3B, 4) showed that Denmark (9, 95%CI: 6–11) and Turkey (27, 95%CI: 14–40) had significantly lower prevalence compared to other European countries, followed by Finland (33, 95%CI: 23–43), Germany (35, 95%CI: 29–41), and Italy (39, 95%CI: 35–44) at a relatively lower level. However, these three countries did not differ significantly from the overall level (Figures 3B, 4). Among PLWH, France had the highest prevalence of NAFLD (61, 95%CI: 47–76), followed by Greece (55, 95%CI, 46–64) and Switzerland (51, 95%CI, 46–56) (Figures 3B, 4). There were no differences in prevalence between other countries and the overall level. Comparing different regions in Europe, the lowest prevalence was found in Northern Europe (24, 95%CI, 4–44), and the highest was in Western Europe (55, 95%CI, 30–80) but the differences between regions in Europe were not statistically significant (Figures 3C, 4). Two multicenter studies from Belgium, France, and Germany showed a high level of prevalence (68, 95%CI, 57–79) for NAFLD (Figures 3D, 4).

It is important to note that, consistent with the findings of Kalligeros, et al.’s meta-analysis, the incidence of NAFLD may be influenced by the choice of diagnostic methods and patient selection. For example, in Lemoine, et al.’s study, if only individuals with persistently elevated liver transaminases were included, the prevalence of NAFLD may be overestimated (39). Another study by Lemone, et al. involving 39 individuals and utilizing high-sensitivity MRI-PDFF for diagnosis, may yield a higher prevalence compared to studies employing other diagnostic methods (40). Therefore, while our overall prevalence results align with Kalligeros, et al.’s meta-analysis, it is crucial approach these results objectively and prudently. To investigate the sources of heterogeneity, we conducted subgroup analyses based on diagnostic methods (Supplementary Figure S6) and meta-regression analyses (Table 2). The subgroup based on diagnostic methods yielded the following prevalence rates for NAFLD: transient elastography (TE) with continuous attenuation parameters (CAP), 46% (95%CI 38–55); ultrasound (U/S), 54% (95%CI 38–70); computed tomography (CT), 26% (95%CI 5–47); hepatic steatosis index (HSI) 60% (95%CI 2–118); magnetic resonance imaging-proton density fat fraction (MRI-PDFF), 42% (95%CI −2–0.86); 1H-magnetic resonance spectroscopy (1H-MRS), 34% (95%CI 22–44); OWLiver Test, 34% (95%CI 17–52); liver biopsy, 63% (95%CI 31–96). From these results, it is evident that liver biopsy has the highest prevalence, which may be attribute to the fact that patients willing to undergo invasive diagnostic methods, such as liver biopsy, are often those with more severe conditions. Meta-regression analyses (Table 2) further revealed that no matter whether the selected independent variable was diagnostic methods (*p* = 0.000), nations (*p* = 0.003), or regions (*p* = 0.001), the resulting *p*-values were all less than 0.05. This also confirmed that both diagnostic methods and different populations contribute as influencing factors to the variable in prevalence.

**Table 2:** Meta-regression for the prevalence of NAFLD, NASH, and liver fibrosis with diagnostic methods, nations, and regions.

		Coef.	Std. Err.	*z*	*P*>|z|	[95%Conf. interval]	
NAFLD	methods	0.0085515	0.0139566	0.61	0.540	−0.018803	0.0359059
	_cons	0.4553941	0.0515264	8.84	0.000	0.3544072	0.5563871
	nation	0.0012587	0.0014733	0.85	0.393	−0.0016289	0.0041463
	_cons	0.0176163	0.0060236	2.92	0.003	0.0058102	0.0294224
	region	0.0012196	0.0030381	0.40	0.688	−0.0047349	0.0071741
	_cons	0.0209255	0.0062449	3.35	0.001	0.0086856	0.0331653
NASH	methods	−0.1106603	0.1104162	−1.00	0.316	−0.327072	0.1057514
	_cons	0.4077311	0.0955762	4.27	0.000	0.2204051	0.5950571
	nation	−0.0877713	0.0330866	−2.65	0.008	−0.1526198	−0.0229229
	_cons	0.5535455	0.0979983	5.65	0.000	0.3614723	0.7456186
	region	−0.0053398	0.0632605	−0.08	0.933	−0.1293281	0.1186486
	_cons	0.3691104	0.1423461	2.59	0.010	0.0901172	0.6481035
Fibrosis	methods	0.0295777	0.0111315	2.66	0.008	0.007603	0.0513952
	_cons	0.0986436	0.0188137	5.24	0.000	0.0617695	0.1355177
	nation	0.0056131	0.0053840	1.04	0.297	−0.0049393	0.0161655
	_cons	0.1072774	0.0289034	3.71	0.000	0.0506277	0.163927
	region	−0.024965	0.0094683	−2.64	0.008	−0.0435226	−0.0064074
	_cons	0.2022208	0.0314995	6.42	0.000	0.140483	0.2639586

### 3.5 Prevalence of NASH

**Figure 5:**
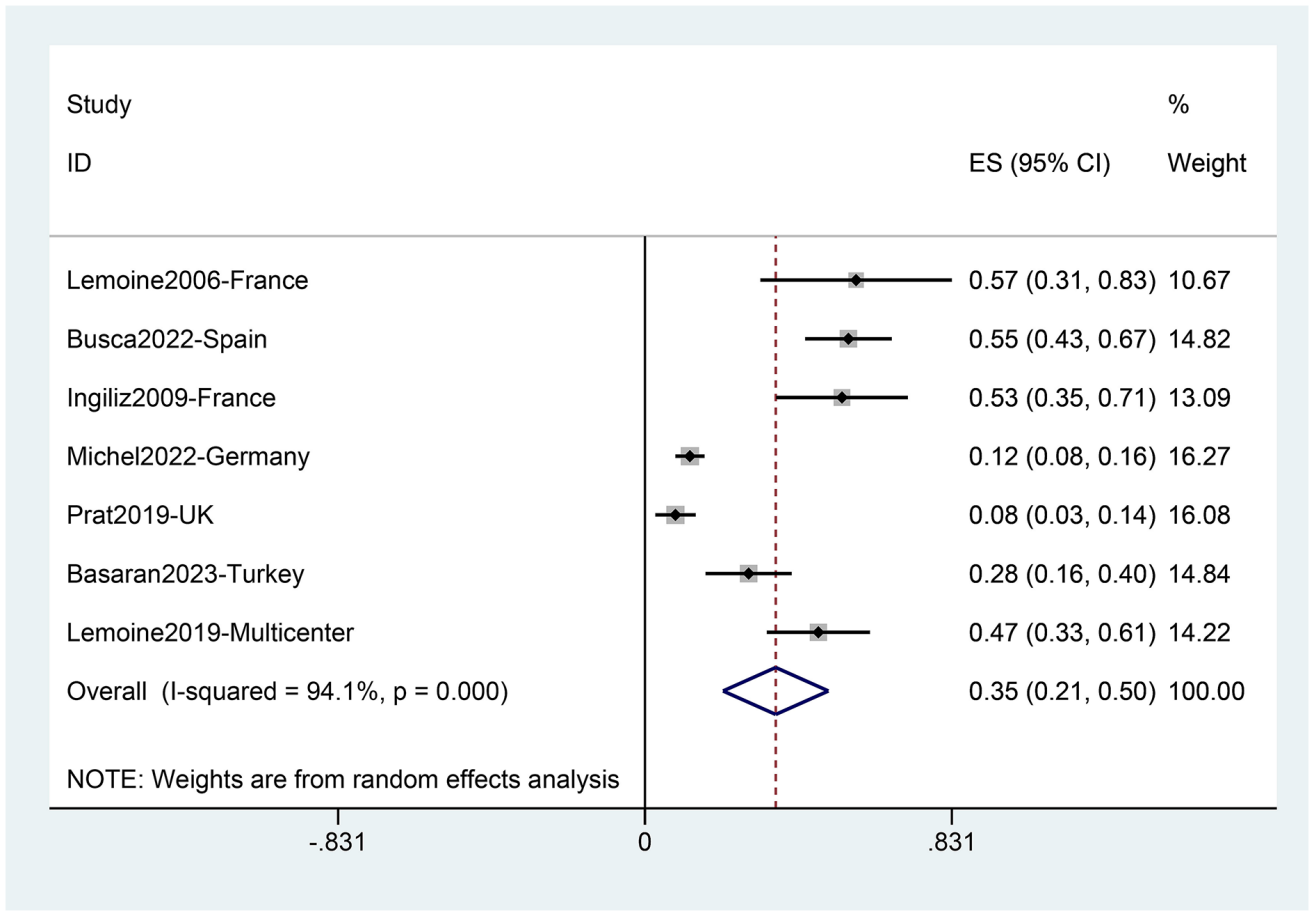
Prevalence of NASH in PLWH in Europe.

The analysis comprised 579 patients from seven studies (31, 33, 35, 39, 43, 46, 52). In the European PLWH, the overall prevalence of NASH was found to be 35% (95%CI: 21–50) (Figure 5). Subgroup analysis based on countries revealed that the lowest NASH prevalence was observed in the United Kingdom (8, 95%CI: 3–14) and Germany (12, 95%CI: 8–16), while the highest rates were observed in France (55, 95%CI: 40–69) and Spain (55, 95%CI: 43–67), with significant differences between them and overall prevalence (Table 3). When comparing different regions in Europe, Central Europe had the lowest prevalence (12, 95%CI: 8–16), showing significant differences compared to other regions. Southern Europe had the highest prevalence (55, 95%CI: 44–64), significantly higher than other regions (Table 3). The prevalence of NASH in the multicenter study (47, 95%CI: 33–61) was not significantly higher than the overall level (35, 95%CI: 21–50) (Figure 5).

**Table 3:** Prevalence of NASH in PLWH mono-infection in Europe subgroup analysis by nation and region.

		Sample size	Pooled prevalence (95%CI)	*p*-value
Nation	Overall analysis	579	35% (21%–50%)	0.000
	France	44	55% (40%–69%)	0.000
	Spain	69	55% (43%–67%)	0.000
	Germany	263	12% (8%–16%)	0.003
	UK	97	8% (3%–14%)	0.000
	Turkey	57	28% (16%–40%)	0.000
	Multicenter (Belgium, France, Germany)	49	47% (33%–61%)	0.000
Region	Overall analysis	579	35% (21%–50%)	0.000
	West Europe	111	31% (−17%–79%)	0.000
	South Europe	99	55% (45%–64%)	0.000
	Central Europe	263	12% (8%–16%)	0.003
	East Europe	57	28% (16%–40%)	0.000
	Multicenter (Belgium, France, Germany)	49	47% (33%–61%)	0.000

Furthermore, subgroup analysis based on different diagnostic methods yielded the following prevalence for NASH: biopsy, 43% (95%CI 18–69); fibroscan-AST score (FAST score), 12% (95%CI 8–16); magnetic resonance elastography (MRE), 28% (95%CI 16–40) (Supplementary Figure S7). Similar to NAFLD, the biopsy group had the highest prevalence. Meta-regression analyses revealed that different diagnostic methods (*p* = 0.001), different nations (*p* = 0.000), and different regions (*p* = 0.010) all contributed as influencing factors to the variable in NASH prevalence (Table 2).

### 3.6 Prevalence of fibrosis

**Figure 6:**
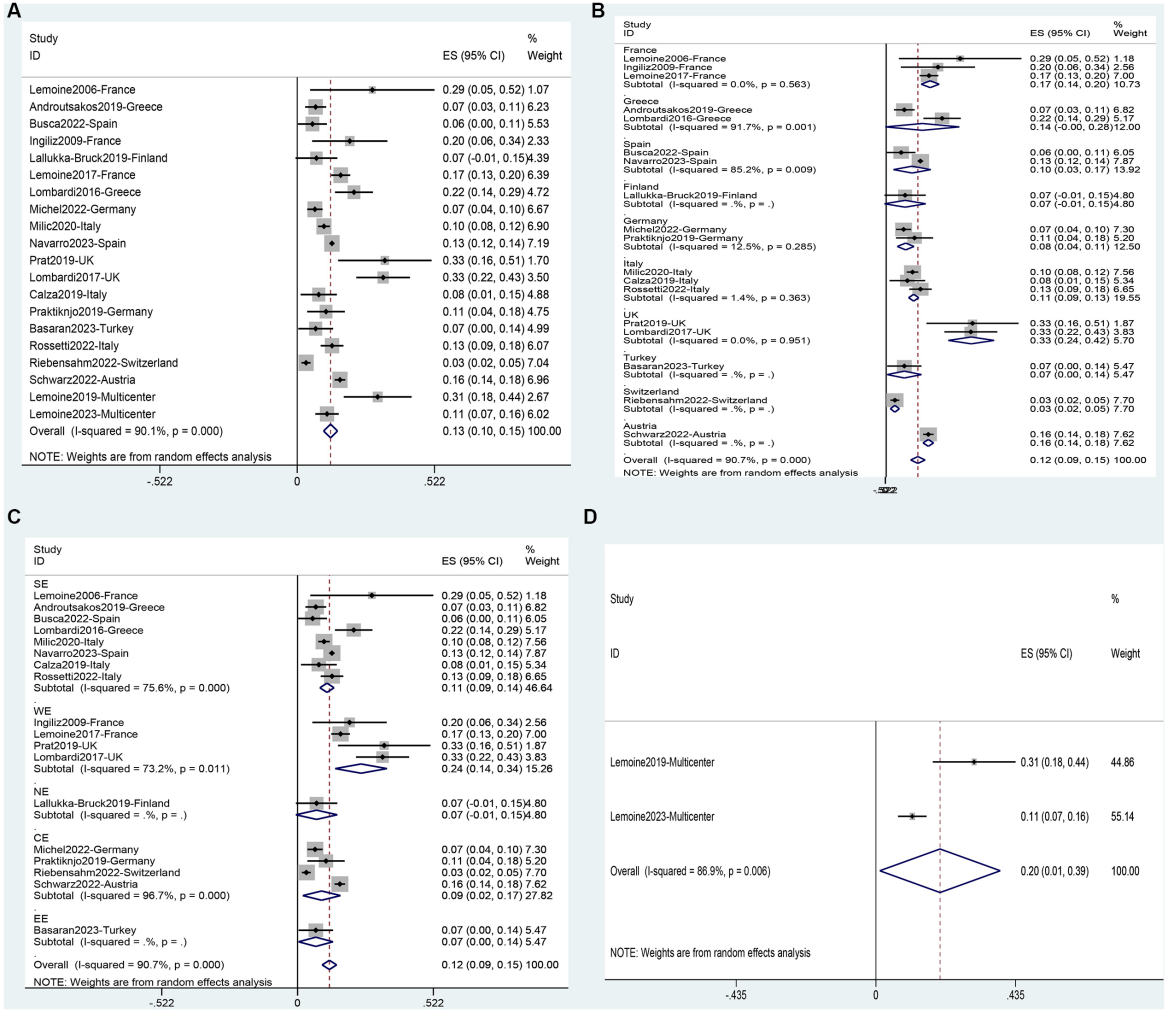
Prevalence of Fibrosis in PLWH in Europe: **(A)**, and subgrouped by nation **(B)**, region **(C)**, and multicenter **(D)**.

This analysis encompassed 916 patients from twenty studies (30, 31, 33, 35, 37–46, 48, 49, 52–54, 59). The overall prevalence of fibrosis in the population was found to be 13% (95%CI: 10–15) (Figure 6A). Subgroup analysis based on countries revealed that Switzerland (3, 95%CI: 2–5) and Germany (8, 95%CI: 4–11) had significantly lower prevalence compared to the overall prevalence. On the other hand, the United Kingdom (33, 95%CI: 24–42), France (17, 95%CI: 14–20), and Austria (16, 95%CI: 14–18) had a significantly higher prevalence than the overall prevalence (Figure 6B). When comparing different regions in Europe, Western Europe (24, 95%CI: 14–34) had a significantly higher prevalence compared to the overall level, while there was no significant difference in the prevalence between other regions in Europe (Figure 6C), including the multicenter studies and the overall level (Figure 6D).

The prevalence of liver fibrosis obtained through subgroup analysis using different diagnostic methods were as follows: fibroscan 10% (95%CI 17–13); NFS, 13% (95%CI 9–16); APRI, 24% (95%CI 8–40), MRE, 7% (95%CI 2–12); and biopsy, 28% (95%CI 20–36) (Supplementary Figure S8). These results showed similarities with the outcome of NAFLD and NASH, where the biopsy group had the highest prevalence. Meta-regression analyses (Table 2) revealed that different diagnostic methods (*p* = 0.000), nations (*p* = 0.000), and regions (*p* = 0.000) were all significant influencing factors for the prevalence of liver fibrosis.

### 3.7 Risk factors for NAFLD

**Figure 7:**
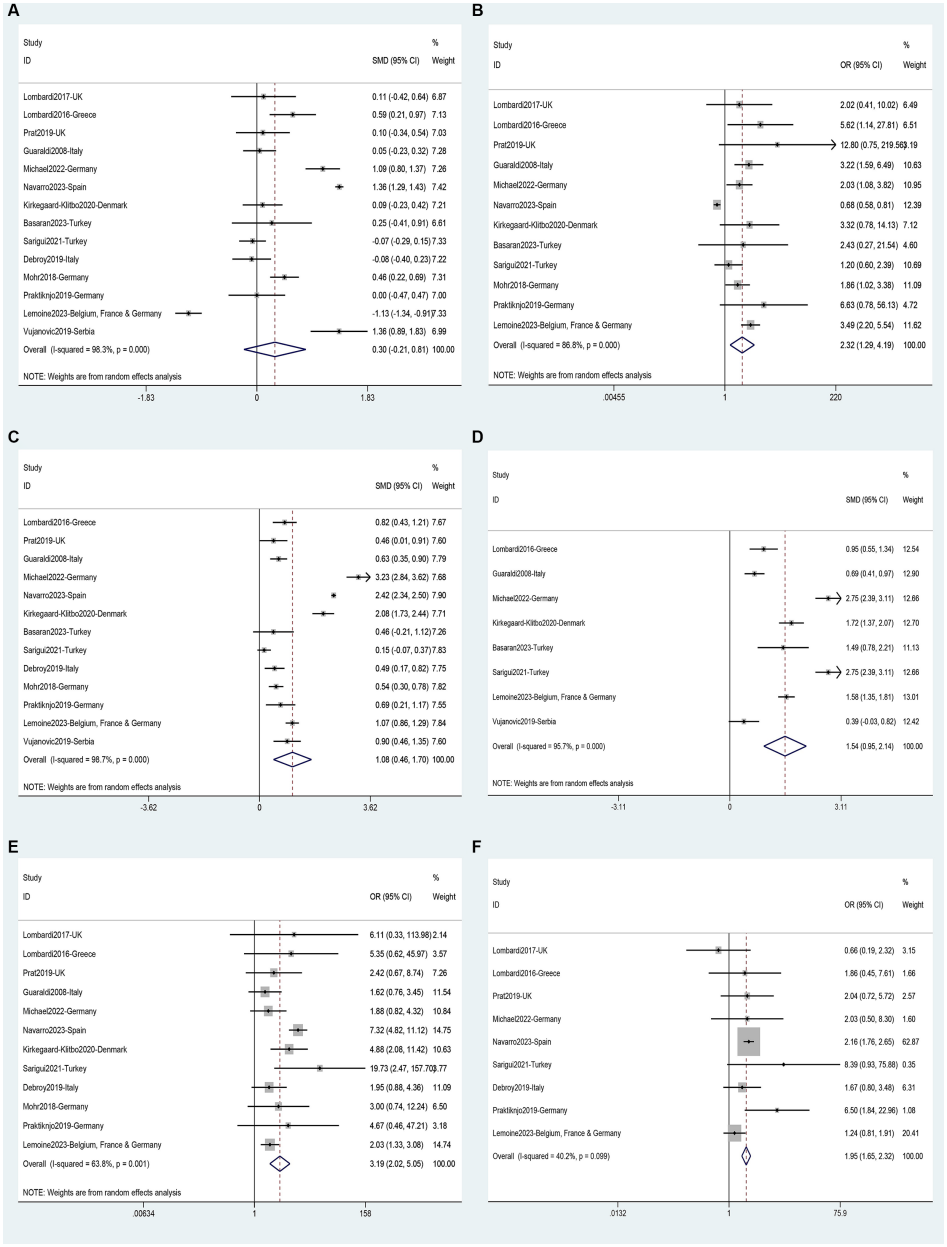
Forest plot for NAFLD risk factors: **(A)** Age, **(B)** Male gender, **(C)** BMI, **(D)** Waist circumference, **(E)** Diabetes, **(F)** Hypertension.

**Figure 8:**
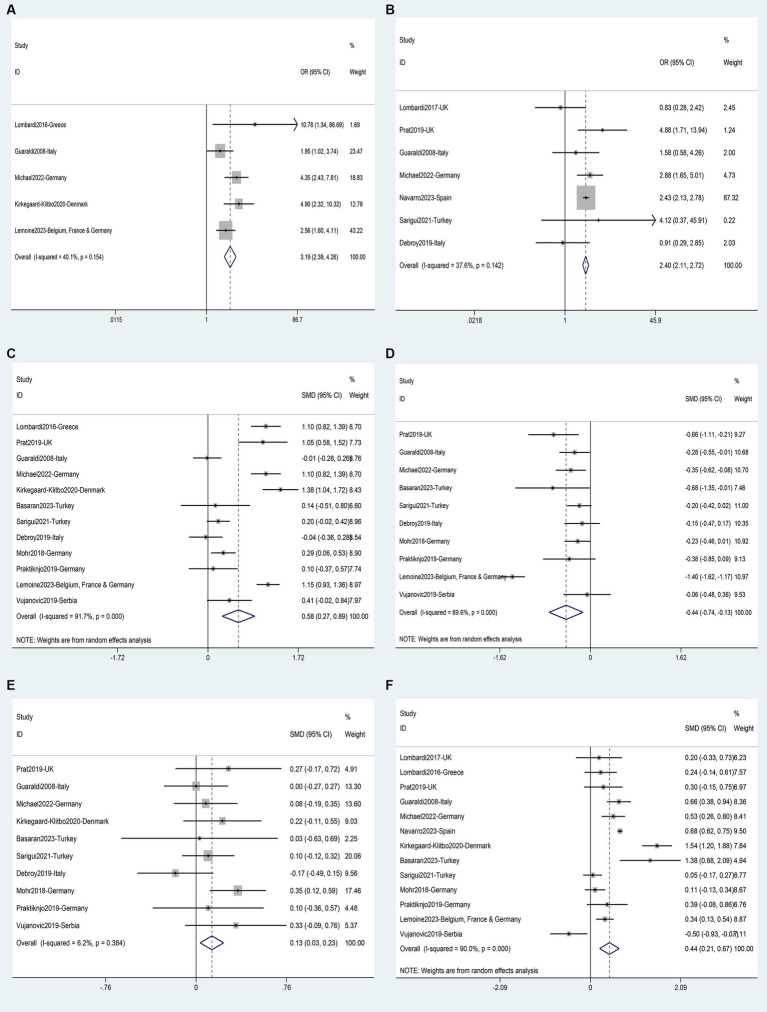
Forest plot for NAFLD risk factors: **(A)** Metabolic syndrome, **(B)** Dyslipidemia, **(C)** Triglycerides, **(D)** HDL, **(E)** LDL, **(F)** ALT.

**Figure 9:**
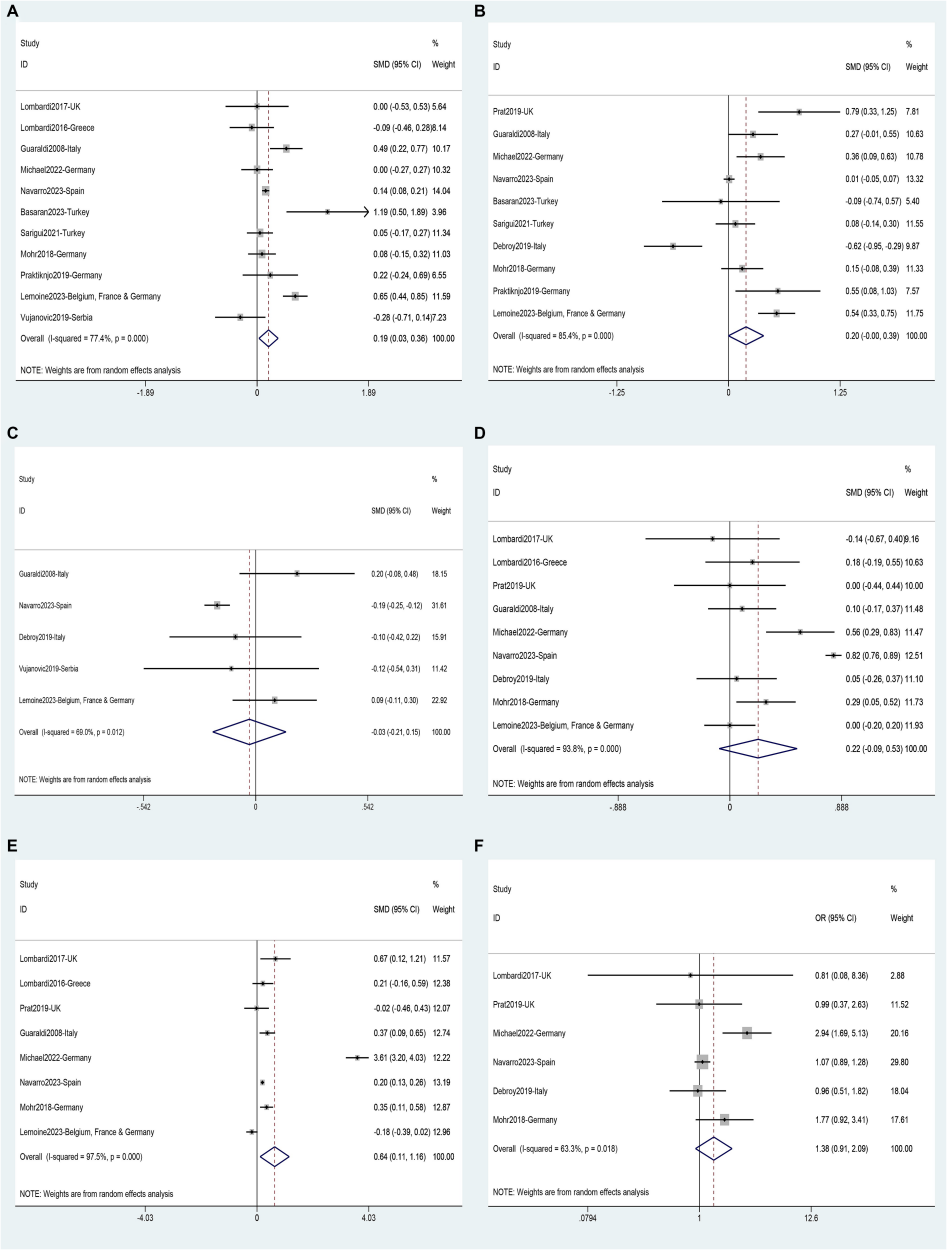
Forest plot for NAFLD risk factors: **(A)** AST, **(B)** CD4 count, **(C)** CD4 nadir, **(D)** Years of HIV infection, **(E)** Years on antiretroviral therapy, **(F)** Undetectable HIV viral load.

Data on risk factors for NAFLD in HIV mono-infected patients was obtained from fourteen studies (11, 36, 40–43, 45–47, 49, 52, 60, 62, 63) involving 7,444 individuals. Using these data, a meta-analysis was conducted on 18 variables (Figures 7–9). Male gender (OR 2.32, 95%CI 1.29–4.19, *p* = 0.005) (Figure 7B), BMI (SMD 1.08, 95%CI 0.46–1.70, *p* = 0.001) (Figure 7C), waist circumference (SMD 1.54, 95%CI 0.95–2.14, *p* = 0.000) (Figure 7D), Diabetes (OR 3.19, 95%CI 2.02–5.05, *p* = 0.000) (Figure 7E), Hypertension (OR 1.95, 95%CI 1.65–2.32, *p* = 0.000) (Figure 7F), metabolic syndrome (OR 3.19, 95%CI 2.39–4.26, *p* = 0.000) (Figure 8A), dyslipidemia (OR 2.40, 95%CI 2.11–2.72, *p* = 0.000) (Figure 8B), triglycerides (SMD 0.58, 95%CI 0.27–0.89, *p* = 0.000) (Figure 8C), HDL (SMD -0.44, 95%CI -0.74—0.13, *p* = 0.005) (Figure 8D), LDL (SMD 0.13, 95%CI 0.03–0.23, *p* = 0.008) (Figure 8E), ALT (SMD 0.44, 95%CI 0.21–0.67, *p* = 0.000) (Figure 8F), AST (SMD 0.19, 95%CI 0.03–0.36, *p* = 0.019) (Figure 9A), and years on antiretroviral therapy (SMD 0.64, 95%CI 0.11–1.16, *p* = 0.017) (Figure 9E) were found to be associated with an increased risk of NAFLD (Supplementary Table S5). However, age (SMD 0.30, 95%CI −0.21–0.81, *p* = 0.253) (Figure 7A), CD4 count (SMD 0.20, 95%CI −0.00–0.39, *p* = 0.052) (Figure 9B), CD4 nadir (SMD −0.03, 95%CI −0.21–0.15, *p* = 0.733) (Figure 9C), years of HIV infection (SMD 0.22, 95%CI −0.09–0.53, *p* = 0.155) (Figure 9D), undetectable HIV viral load (OR 1.38, 95%CI 0.91–2.09, *p* = 0.125) (Figure 9F) were not significantly associated with the prevalence of NAFLD (Supplementary Table S5).

### 3.8 Risk factors for NASH

**Figure 10:**
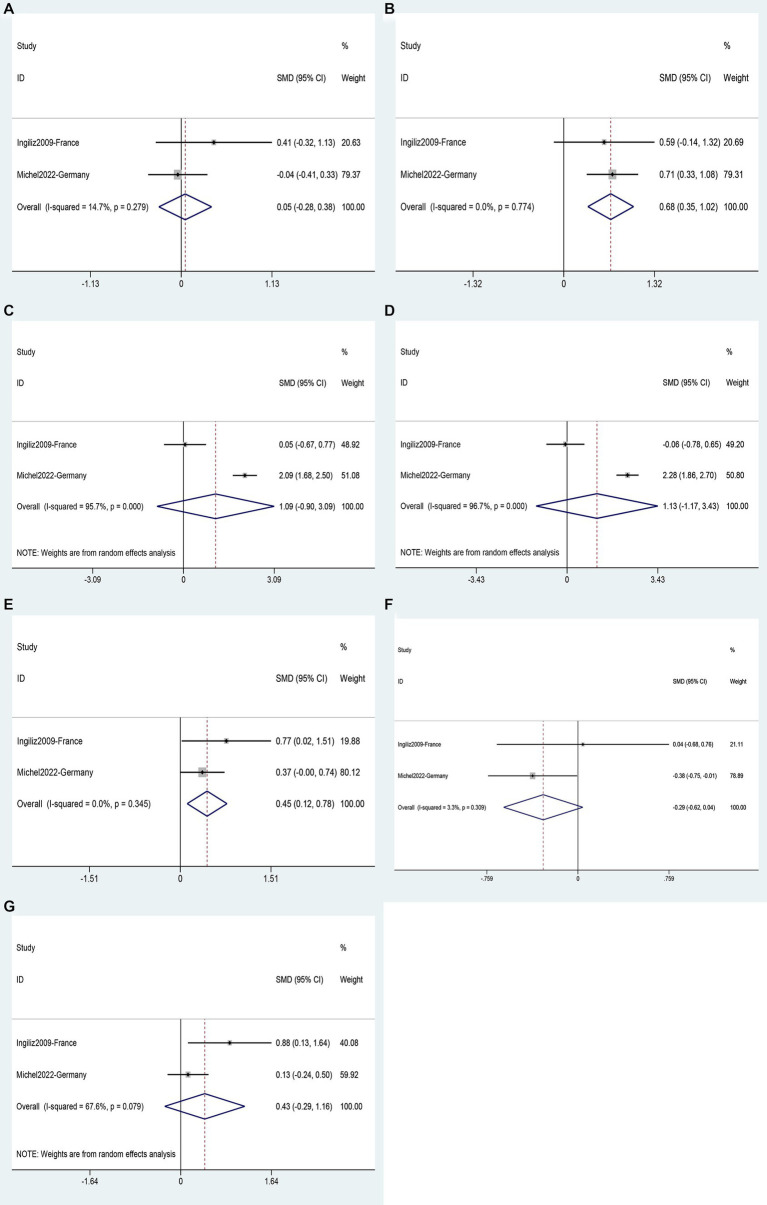
Forest plot for NASH risk factors: **(A)** Age, **(B)** BMI, **(C)** ALT, **(D)** AST, **(E)** Triglycerides, **(F)** Total cholesterol, **(G)** CD4 count.

Two studies (35, 43) involving a total of 293 patients provided data for the analysis of seven possible risk factors for NASH. Body Mass Index (BMI) (SMD 0.68, 95%CI 0.35–1.02, *p* = 0.000) (Figure 10B) and triglycerides (SMD 0.45, 95%CI 0.12–0.78, *p* = 0.008) (Figure 10E) were identified as risk factors for NASH, while age (SMD 0.05, 95%CI −0.28–0.38, *p* = 0.765) (Figure 10A), ALT (SMD 1.09, 95%CI −0.90–3.09, *p* = 0.283) (Figure 10C), AST (SMD 1.13, 95%CI −1.17–3.43, *p* = 0.336) (Figure 10D), total cholesterol (SMD −0.29, 95%CI −0.62–0.04, *p* = 0.085) (Figure 10F) and CD4 count (SMD 0.43, 95%CI −0.29–1.16, *p* = 0.239) (Figure 10G) were not supported as risk factors for NASH (Supplementary Table S5).

### 3.9 Risk factors for fibrosis

**Figure 11:**
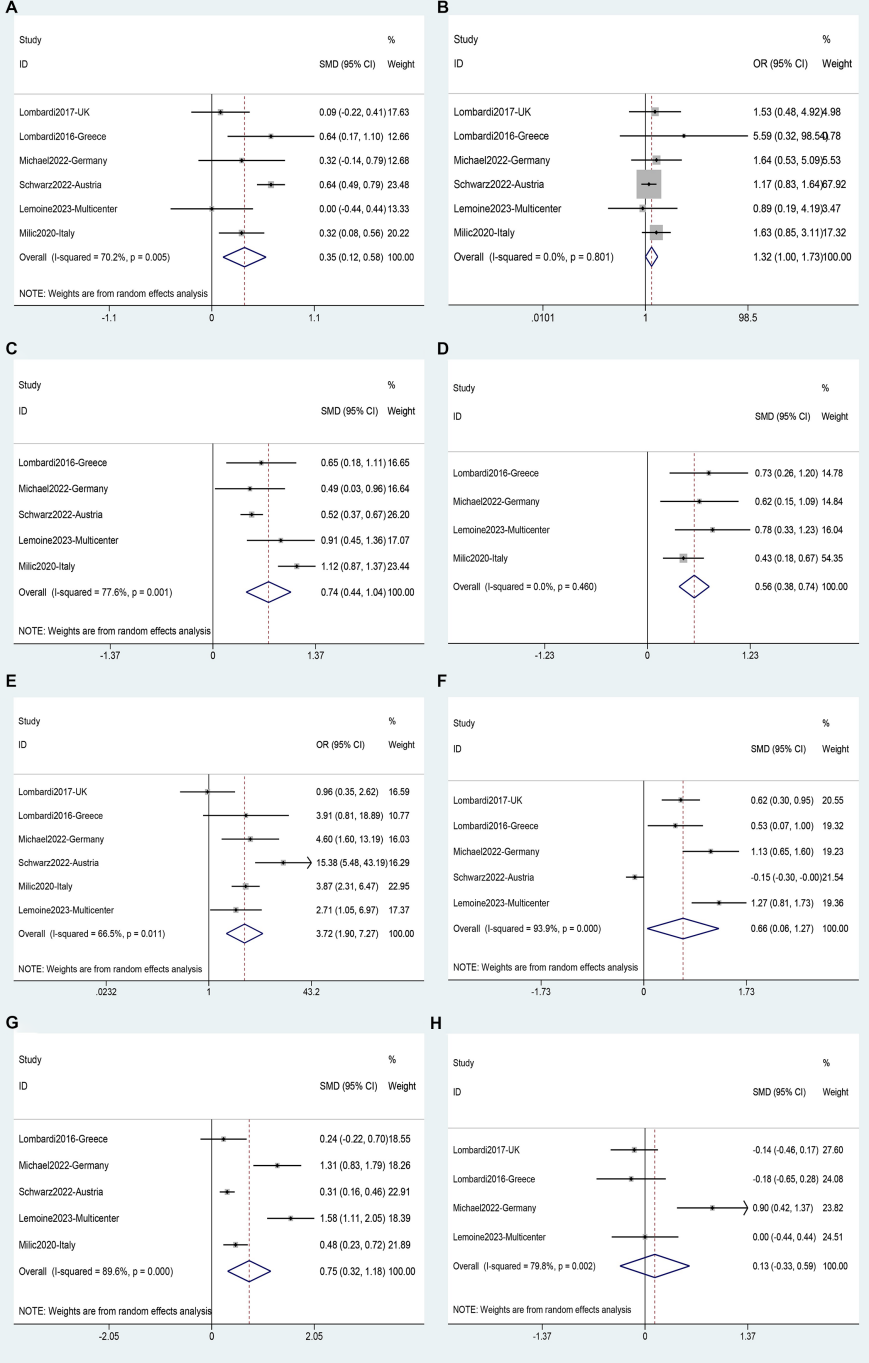
Forest plot for Liver fibrosis risk factors: **(A)** Age, **(B)** Male gender, **(C)** BMI, **(D)** Waist circumference, **(E)** Diabetes, **(F)** ALT, **(G)** AST, **(H)** Years of HIV infection.

The analysis of risk factors for liver fibrosis was based on data from 6 studies (40–44, 59) involving 2,734 patients. Fibrosis was found to be associated with age (SMD 0.35, 95%CI 0.12–0.58, *p* = 0.003) (Figure 11A); BMI (SMD 0.74, 95%CI 0.44–1.04, *p* = 0.016) (Figure 11C), waist circumference (SMD 0.56, 95%CI 0.38–0.74, *p* = 0.000) (Figure 11D); diabetes (OR 3.72, 95%CI 1.90–7.27, *p* = 0.111) (Figure 11E); ALT (SMD 0.66, 95%CI 0.06–1.27, *p* = 0.000) (Figure 11F), and AST (SMD 0.75, 95%CI: 0.32–1.18, *p* = 0.000) (Figure 11G). Male gender (OR 1.32, 95%CI 1.00–1.73, *p* = 0.111) (Figure 11B), and years of HIV infection (SMD 0.13, 95%CI −0.33–0.59, *p* = 0.640) (Figure 11H) were not observed to be associated with Fibrosis (Supplementary Table S5).

## 4 Discussion

This study represents the first meta-analysis of the prevalence of NAFLD, NASH, and fibrosis, along with associated risk factors, in PLWH across European countries. We meticulously excluded studies that involved concurrent infections with hepatitis viruses such as HCV and HBV, as well as those with high alcohol intake. In total, we included thirty-six studies in our analysis. It is important to note that different diagnostic methods can have some impact on the positivity rate for NAFLD, but this variability is inevitable (23, 24). To mitigate heterogeneity, we adopted a methodology similar to previous studies, prioritizing diagnostic results obtained through ultrasound examinations or other imaging techniques (23). Moreover, for studies with excessive heterogeneity, we rigorously applied a random-effects model in our analysis (23).

Based on the research data from thirty-six studies, encompassing 7,893 patients, our analysis revealed that the overall prevalence of NAFLD among European individuals with HIV mono-infection stands at a substantial 42%. In the adult population, this figure reaches 43%. Notably, this finding aligns perfectly with the 42.79% reported by Kalligeros et al. in their recent study (23). While they included eighteen studies from Europe, only half the number included in our analysis, the consistency in research outcomes is remarkable. This further underscores the reliability and stability of the study results (23).

The reasons behind the high prevalence of NAFLD in European PLWH are not yet clear. However, it is speculated that this may be related to variations in the distribution of NAFLD susceptibility genes among different ethnic groups. For instance, variations in the PNPLA3 gene, which encodes a lipid-editing enzyme, have been shown to significantly impact liver fat content and susceptibility to NAFLD. This variation is more common in European Caucasians and Hispanics than in African populations (65, 66). Recently, the TM6SF2 gene has also been found to play a significant role in the development and progression of NAFLD, with the non-synonymous E167K mutation within it considered one of the strongest genetic risk factors for NAFLD development (67). Additionally, Mancia et al. discovered that the MBOAT7 gene variant rs641738 may be associated with the occurrence and progression of NAFLD in Europeans. However, further research is necessary to investigate the distribution of these genetic variations across different populations. A recent report has highlighted the spread of a highly virulent strain of HIV in Europe (68). Nevertheless, given that undetectable HIV RNA viral load were not linked to a decreased likelihood of NAFLD, NASH and liver fibrosis, there is no enough evidence to suggest that viral virulence is a risk factor.

According to subgroup analyses conducted at the national and regional levels, the trends in the prevalence of NAFLD, NASH, and liver fibrosis among European PLWH are not entirely consistent. For instance, the prevalence of NAFLD in Switzerland is significantly higher than the overall level, but the prevalence of fibrosis is notably lower than the overall rate. This inconsistency in trends may be related to specific genetic, and environmental factors, and medical practices in different countries and regions. In Northern Europe, countries like Denmark and Finland have relatively low rates of NAFLD and fibrosis among PLWH compared to the European average. This might reflect successful practices in the prevention and treatment of NAFLD and fibrosis in the Northern European region or the protective role of specific genetic and environmental factors in this area. Conversely, in Western Europe, France stands out with significantly higher rates of NAFLD, NASH, and fibrosis compared to the overall average. However, it is worth noting that the prevalence in France were obtained using the biopsy diagnostic method, which is invasive and usually accepted by patients with more severe conditions. Therefore, the results obtained may be biased towards higher prevalence. This is further supported by the results of the subgroup analyses (Supplementary Figures S6–S8) and meta-regression analysis (Table 2). As a result, these results should be interpreted with caution. However, France boasts the highest HIV treatment coverage in Europe. Antiretroviral therapy (ART) may contribute to a likelihood of NAFLD-related metabolic abnormalities among patients (69, 70). While genetic and environmental factors may play a role, the impact of ART treatment cannot be overlooked as a potential contributing factor.

This study has uncovered that the duration of ART is the only variable related to HIV in the PLWH population that poses a risk factor for NAFLD. All other risk factors align with those found in the general population (71–73). As for NASH and liver fibrosis, no risk factors different from the general population have been found. This emphasizes the need to follow largely similar preventive strategies for promoting overall health in PLWH individuals, as is recommended for the general population. However, the impact of ART on NAFLD should not be overlooked. Early protease inhibitors, such as Lopinavir and Atazanavir, are known to cause moderate to severe liver toxicity (74). Nevertheless, Darunavir is currently the only commonly used protease inhibitor for HIV treatment, which is utilized as a booster in combination with Ritonavir or Cobicistat. Notably, it has significantly reduced liver toxicity compared to the first-generation protease inhibitors (75, 76). Pires et al. have suggested that NAFLD induced by antiretroviral therapy may primarily be associated with D-analogs (e.g., Didanosine/ddI, Stavudine/d4T, and Zalcitabine/ddC) (70). Nevertheless, these medications are not commonly used in contemporary HIV treatment in Europe as well. A recent five-year study from Germany found that the duration of treatment with second generation nucleoside reverse transcriptase inhibitors, Tenofovir Alafenamide (TAF)/Emtricitabine and second generation integrase inhibitors, Dolutegravir (DTG), was associated with increased risk of NAFLD in HIV patients (32). However, the third generation of Integrase inhibitors, such as Lenacapvir, have minimal impact on cytochrome P450 enzymes, leading to very rare instances of liver toxicity (77). It remains unclear whether second-generation NRTIs and Integrase inhibitors are the primary ARTs responsible for causing NAFLD in PLWH in Europe. Furthermore, since most HIV patients are typically exposed to multiple types of ART drugs, further research is required to investigate this matter in the future.

NASH represents a more severe progression of NAFLD, involving liver cell damage during the inflammatory process and carrying an increased risk of further developing liver fibrosis, cirrhosis, and liver cancer (78, 79). It is estimated that the average incidence of NASH in the general population is 3.5–5% (80). However, PLWH who have NAFLD are more prone to developing NASH than NAFLD patients in the general population (81). Our analysis based on seven studies indicated that the prevalence of NASH in the European PLWH is 35%. It should be emphasized that, although liver biopsy is the most accurate method for diagnosing NASH, only patients with more severe conditions would undergo invasive liver biopsy examinations. Therefore, the prevalence of NASH obtained from liver biopsies could be overestimated, as it represents a group that is enriched due to the inherent selection bias associated with an intrusive diagnostic procedure. Nevertheless, the results obtained are comparable to those of previously published meta-analyses with the same inclusion criteria, thus rendering them significant. For instance, compared with the results of a meta-analysis on NASH prevalence among liver biopsy patients completed by Maurice et al. (24), the prevalence of NASH in European PLWH (35%) is lower than the global prevalence (42%) reported by Maurice et al. This regional difference may be associated with specific genetic factors within the European population (82) or may be attributed to early screening, diagnosis, and treatment of NAFLD. A Finnish study conducted genome-wide analysis on 8,434 European ancestry NAFLD patients and identified five susceptibility genes, indicating that NAFLD could be a kind of disease caused by multiple factors related to genetic variation (82). However, there is currently a lack of information about the distribution of susceptibility genes among different population worldwide, thus requiring future studies to be more comprehensive and in-depth. In April 2016, the European Association for the Study of the Liver released NAFLD clinical practice guidelines, which provided thirty-eight recommendations for the screening, diagnosis, treatment, and follow-up of NAFLD in European countries (83). Effective early intervention and management strategies can help prevent the progression of NAFLD to NASH. Furthermore, our meta-analysis of risk factors for NASH in the PLWH for the first time reveals that BMI and hypertriglyceridemia are associated with the prevalence of NASH in the European PLWH. This suggests that factors related to NASH in the European PLWH may be linked to weight and lipid abnormalities.

Previous studies have suggested that 36.1% of NAFLD patients may experience progressive liver fibrosis (73). Consistent with this, our study showed the prevalence of liver fibrosis in the European PLWH is 13%, which is almost exactly one-third of the NALFD prevalence (42%) in the European PLWH and nearly identical to the prevalence (12%) of fibrosis in the global PLWH as reported by Kallilgeros et al. (23). Similar to NASH, this finding may reflect some positive measures in liver health management and interventions in Europe, as well as other influencing factors that may exist among PLWH in different regions, such as regional genetic differences, more frequent liver function tests, regular health education, easier access to treatment, and support. These factors may contribute to preventing NAFLD from progressing to liver fibrosis, thereby reducing the incidence of liver fibrosis.

Through the analysis of liver fibrosis risk factors in the European PLWH, we found a significantly increased incidence of liver fibrosis associated with age, BMI, waist circumference, diabetes, ALT, AST, and years of HIV infection. This suggests that metabolic abnormalities, especially obesity and diabetes, play a significant role in the development of liver fibrosis. This conclusion is consistent with previous findings on risk factors for liver fibrosis (24). Furthermore, our study did not find a correlation between the duration of HIV infection and liver fibrosis, which supports the findings of Maurice’s research (24).

The limitations of this study primarily include the following aspects: (1) Only one study on European adolescents living with HIV was included, and this study only provided data on the prevalence of NAFLD, making comprehensive comparison and analysis difficult. Nonetheless, given the considerably lower prevalence of NAFLD among adolescents compared to adults, the focus of research remains on adults. (2) Most of the included studies were cross-sectional studies, which limited the ability of this study to infer causal relationships. (3) From the analysis results of this study, it can be observed that the duration of receiving ART treatment in HIV patients is also one of the risk factors for NAFLD. If data on the impact of different types of HAART treatment drugs on NAFLD prevalence were available, it might reveal valuable information. However, due to the lack of relevant data, such an analysis cannot be conducted at this time. (4) There are relatively few studies from Central and Eastern Europe, thereby limiting their representativeness. This will be addressed with updates as more relevant research becomes available in the future.

## 5 Conclusion

In summary, this study conducted a stratified assessment of the prevalence of NAFLD, NASH, and fibrosis, as well as potential risk factors among PLWH in different countries and regions in Europe. The analysis guided the future prevention and treatment of NAFLD and related diseases in PLWH in Europe. It is advisable to implement early and proactive screening and interventions based on the risk factors that may influence the occurrence, progression, and prognosis of NAFLD, as well as the prevalence rates in different European countries and regions. This approach can lead to more targeted public health policies and medical measures, ultimately contributing to a fundamental improvement in the health of PLWH in Europe.

## Data availability statement

The original contributions presented in the study are included in the article/Supplementary material, further inquiries can be directed to the corresponding authors.

## Author contributions

DJ: Writing – original draft, Writing – review & editing, Formal analysis, Methodology, and Project administration. TZ: Conceptualization, Investigation, Writing – review & editing. SJ: Data curation, Investigation, Writing – review & editing. ZC: Data curation, Investigation, Writing – review & editing. BG: Methodology, Software, Writing – review & editing. GL: Project administration, Validation, Writing – review & editing. CZ: Resources, Supervision, Validation, Writing – review & editing.
