# Supplementary material for: Regional variation in NAFLD prevalence and risk factors among people living with HIV in Europe: a meta-analysis
Source: Front Public Health. 2024 Jan 4;11:1295165. doi: 10.3389/fpubh.2023.1295165 (PMC10802187; doi:10.3389/fpubh.2023.1295165)
Supplement: Supplementary file 1 [file Data_Sheet_1.PDF]

# *Supplementary Material*

## 1 Supplementary Tables

**Table S1. Summary of included studies**

| **Author** | **Year** | **Nation** | **TN** | **Study period** | **Study design** | **Population** | **DxM for NAFLD/NASH** | **DxM for Fibrosis** |
|---|---|---|---|---|---|---|---|---|
| **Lemoine** | 2006 | France | 14 | 2003.01-2005.05 | Cross-sectional | Adult | NAFLD: Biopsy<br><br>NASH: Biopsy | Biopsy |
| **Androutsakos** | 2019 | Greece | 157 | 2015.01-0217.12 | Cross-sectional | Adult | n/a | TE≥9kPa |
| **Bischoff** | 2021 | Germany | 319 | 2013.08-2018.12 | Longitudinal prospective observational | Adult | NAFLD: CAP≥238dB/m | n/a |
| **Busca** | 2022 | Spain | 69 | 2017.01-2018.06 | Cross-sectional | Adult | NAFLD: US<br><br>NASH: Biopsy | NFS＞-1.45 |
| **Guaraldi** | 2008 | Italy | 225 | 2006.01-2007.06 | Cross-sectional | Adult | NAFLD: CT | n/a |
| **Guaraldi** | 2011 | Italy | 103 | 2007.04-2008.04 | Cross-sectional | Adult | NAFLD: US | n/a |
| **Ingiliz** | 2009 | France | 30 | n/a | Cross-sectional | Adult | NAFLD: Biopsy<br><br>NASH: Biopsy | Biopsy |
| **Kirkegaard-Klitbo** | 2020 | Denmark | 453 | 2015.03-2016.11 | Cross-sectional | Adult | NAFLD: CT | |
| **Lallukka-Bruck** | 2019 | Finland | 41 | n/a | Longitudinal prospective observational | Adult | NAFLD:$^1$H-MRS(LEAF＞5.56%) | MRE＞3.62kPa |

| | | | | | | | | |
|---|---|---|---|---|---|---|---|---|
| **Lemoine** | 2017 | France | 405 | 2011.01-2012.12 | Cross-sectional | Adult | n/a | LSM≥7.1 kPa |
| **Lemoine** | 2019 | MC | 49 | n/a | Cross-sectional | Adult | NAFLD: Biopsy<br>NASH: Biopsy | Biopsy |
| **Lemoine** | 2023 | MC | 402 | 2014.03-2015.11 | Prospective observational | Adult | NAFLD: MRI-PDFF≥5% | LSM≥9.6 kPa |
| **Lombardi** | 2016 | Greece | 125 | NA | Cross-sectional | Adult | NAFLD: US | LSM＞7.4kPa |
| **Lombardi** | 2017 | UK | 66 | 2014.01-2014.12 | Cross-sectional | Adult | NAFLD: US | APRI＞0.5 |
| **Michel** | 2022 | Germany | 282 | 2018-2021 | Cross-sectional | Adult | NAFLD: CAP≥275dB/m<br>NASH: FAST score＞0.35 | LSM≥8.2 kPa |
| **Milic** | 2020 | Italy | 707 | 2018.06-2019.05 | Cross-sectional | Adult | NAFLD: CAP≥248dB/m | LSM≥7.1 kPa |
| **Navarro** | 2023 | Spain | 4798 | 2004.01-2019.12 | Prospective cohort study | Adult | NAFLD: HSI≥36 | NFS＞-1.455 |
| **Prat** | 2019 | UK | 97 | 2000.01-2017.06 | Cross-sectional | Adult | NAFLD: Biopsy<br>NASH: Biopsy | Biopsy |
| **Vujanovic** | 2019 | Serbia | 88 | 2016.09-2018.04 | Cross-sectional | Adult | NAFLD: US | n/a |
| **Calza** | 2019 | Italy | 61 | 2014.06-2016.05 | Longitudinal prospective observational | Adult | NAFLD: CAP≥260dB/m | LSM≥8.2 kPa |
| **Praktiknjo** | 2019 | Germany | 73 | n/a | Cross-sectional | Adult | NAFLD: CAP≥253dB/m | Fibroscan |

| | | | | | | | | |
|---|---|---|---|---|---|---|---|---|
| **Macias** | 2017 | Spain | 39 | 2014.02-2015.09 | Longitudinal prospective observational | Adult | NAFLD: CAP≥238dB/m | n/a |
| **Macias** | 2016 | Spain | 326 | 2011.11-2013.10 | Longitudinal prospective observational | Adult | NAFLD: CAP≥238dB/m | n/a |
| **Basaran** | 2023 | Turkey | 57 | n/a | Cross-sectional | Adult | NAFLD: MRI-PDFF＞5%<br>NASH: MRE 2.5-2.9kPa | MRE＞2.9kPa |
| **Rossetti** | 2022 | Italy | 234 | 2014.01-2020.02 | Cross-sectional | Adult | n/a | TE＞6.65kPa |
| **Riebensahm** | 2022 | Switzerland | 416 | 2019.11-2021.08 | Cross-sectional | Adult | NAFLD:CAP≥248dB/m | LSM≥7.1kPa |
| **Moreno-Perez** | 2018 | Spain | 72 | 2009.03-2010.10 | Cross-sectional | Adult | NAFLD:$^{1}$H-MRS＞5% | n/a |
| **Curran** | 2020 | Spain | 29 | n/a | Longitudinal prospective observational | Adult | NAFLD: OWLiver Test | n/a |
| **Hanttu** | 2021 | Finland | 43 | 2018.02-2019.05 | Cross-sectional | Adult | NAFLD:MRS | n/a |
| **Austermann** | 2017 | Germany | 65 | 2013.09-2016.03 | Longitudinal prospective observational | Adult | NAFLD:CAP≥260dB/m | n/a |
| **Schwarz** | 2022 | Austria | 1272 | 2013.09-2016.03 | Longitudinal prospective observational | Adult | n/a | NFS＞-1.455 |
| **Sarigui** | 2021 | Turkey | 358 | 2015.06-2019.09 | Cross-sectional | Adult | NAFLD: US | n/a |
| **Maric** | 2020 | Serbia | 88 | 2017.05-2018.01 | Cross-sectional | Adult | NAFLD: US | n/a |
| **Debroy** | 2019 | Italy | 169 | 2010.10-2015.02 | Cross-sectional | Adult | NAFLD: CT | n/a |

| | | | | | | | | |
|---|---|---|---|---|---|---|---|---|
| **Mohr** | 2018 | Germany | 289 | n/a | Cross-sectional | Adult | NAFLD:CAP≥ 238dB/m | n/a |
| **Rubio** | 2009 | France | 23 | n/a | Cross-sectional | Adolescent | US | n/a |

Note: TN: Total number; NAFLD: Non-alcoholic fatty liver; NASH: Non-alcoholic steatohepatitis; DxM: Diagnostic method; TE: Transient elastography; CAP: continuous attenuation parameters; US: Ultrasound; $^{1}$H-MRS: $^{1}$H magnetic resonance spectroscopy； LEAF: Liver fat estimation algorithm; LSM: Liver stiffness measurement; MC: Multicenter(Belgium, France, and Germany); MRI-PDFF: Magnetic resonance imaging proton density fat fraction ; UK: The United Kingdom; APRI: Aspartate aminotransferase to platelet ratio index; NFS: NAFLD-fibrosis score; MRS: Magnetic resonance spectroscopy; MRE Magnetic resonance elastography: CT: Computed tomography; FAST score: Fibroscan-AST score; NA: Not available

Table S2. JBI Quality Assessment

| Study | Were the criteria for inclusion in the sample clearly defined | Were the study subjects and the setting described in detail? | Was the exposure measured in a valid and reliable way? | Were objective, standard criteria used for measurement of the condition? | Were confounding factors identified? | Were strategies to deal with confounding factors stated? | Were the outcomes measured in a valid and reliable way? | Was appropriate statistical analysis used? | Overall appraisal |
|---|---|---|---|---|---|---|---|---|---|
| Lemoine2006-France | Y | Y | Y | Y | U | N | Y | Y | 6/8 |
| Androutsakos2019-Greece | Y | Y | Y | Y | Y | Y | Y | Y | 8/8 |
| Bischoff2021-Germany | Y | Y | Y | Y | U | N | Y | Y | 6/8 |
| Busca2022-Spain | Y | Y | Y | Y | Y | Y | Y | Y | 8/8 |
| Guaraldi2008-Italy | Y | Y | Y | Y | Y | Y | Y | Y | 8/8 |
| Guaraldi2011-Italy | Y | Y | Y | Y | Y | Y | Y | Y | 8/8 |
| Ingiliz2009-France | Y | N | Y | Y | Y | Y | Y | Y | 7/8 |
| Kirkegaard-Klitbo2020-Denmark | Y | Y | Y | Y | U | N | N | Y | 5/8 |
| Lallukka-Bruck2019-Finland | Y | Y | Y | Y | U | N | Y | Y | 6/8 |
| Lemoine2017-France | Y | Y | Y | Y | Y | Y | Y | Y | 8/8 |
| Lemoine2019-Belgium, France &Germany | Y | N | Y | Y | Y | Y | Y | Y | 7/8 |
| Lemoine2023-Belgium, France & Germany | Y | N | Y | Y | Y | Y | Y | Y | 7/8 |
| Lombardi2016-Greece | Y | N | Y | Y | Y | Y | Y | Y | 7/8 |
| Lombardi2017-UK | Y | Y | Y | Y | U | N | Y | Y | 6/8 |

| | | | | | | | | | |
|---|---|---|---|---|---|---|---|---|---|
| Michel2022-Germany | Y | Y | Y | Y | U | N | Y | Y | 6/8 |
| Milic2020-Italy | Y | Y | Y | Y | U | N | Y | Y | 6/8 |
| Navarro2023-Spain | Y | Y | Y | Y | U | N | Y | Y | 6/8 |
| Prat2019-UK | Y | Y | Y | Y | Y | Y | Y | Y | 8/8 |
| Vujanovic2019-Serbia | Y | Y | Y | Y | U | N | Y | Y | 6/8 |
| Calza2019-Italy | Y | Y | Y | Y | U | N | Y | Y | 6/8 |
| Praktiknjo2019-Germany | Y | Y | Y | Y | Y | Y | Y | Y | 8/8 |
| Macias2016-Spain | Y | N | Y | Y | Y | Y | Y | Y | 7/8 |
| Macias2017-Spain | Y | Y | Y | Y | U | N | Y | Y | 6/8 |
| Basaran2023-Turkey | Y | N | Y | Y | U | N | Y | Y | 5/8 |
| Rossetti2022-Italy | Y | Y | Y | Y | U | N | Y | Y | 6/8 |
| Riebensahm2022-Switzerland | Y | Y | Y | Y | Y | Y | Y | Y | 8/8 |
| Moreno-Perez2018-Spain | Y | Y | Y | Y | U | N | Y | Y | 6/8 |
| Curran2020-Spain | Y | Y | Y | Y | Y | Y | Y | Y | 8/8 |
| Hanttu2021-Finland | Y | Y | Y | Y | Y | Y | Y | Y | 8/8 |
| Austermann2017-Germany | Y | Y | Y | Y | Y | Y | Y | Y | 8/8 |
| Schwarz2022-Austria | Y | Y | Y | Y | U | N | Y | Y | 6/8 |
| Sarigui2021-Turkey | Y | N | Y | Y | Y | Y | Y | Y | 7/8 |
| Maric2020-Serbia | Y | Y | Y | Y | U | N | Y | Y | 6/8 |
| Debroy2019-Italy | Y | Y | Y | Y | U | N | Y | Y | 6/8 |

| | | | | | | | | | |
|---|---|---|---|---|---|---|---|---|---|
| Mohr2018-Germany | Y | Y | Y | Y | Y | Y | Y | Y | 8/8 |
| Rubio2009-France | Y | Y | Y | Y | Y | Y | Y | Y | 8/8 |

## Table S3. Results of Begg's Test

| Variables | | P-value |
|---|---|---|
| **NAFLD** | | 0.710 |
| **NASH** | | 0.230 |
| **Fibrosis** | | 0.230 |
| **NAFLD risk factors** | Age | 0.010 |
| | Gender | 0.451 |
| | BMI | 0.860 |
| | Waist circumference | 0.893 |
| | Diabetes | 0.086 |
| | Hypertension | 0.348 |
| | Metabolic syndrome | 0.221 |
| | Dyslipidemia | 0.764 |
| | Triglyceride | 0.980 |
| | HDL | 0.820 |
| | LDL | 0.284 |
| | ALT | 0.758 |
| | AST | 0.955 |
| | CD4 count | 0.770 |
| | CD4 nadir | 0.474 |
| | Years of HIV infection | 0.096 |
| | Years on ART | 0.499 |
| | Undetectable HIV viral load | 0.707 |
| **NASH risk factors** | Age | 1.000 |
| | BMI | 0.734 |
| | ALT | 0.734 |
| | AST | 0.734 |
| | TG | 0.734 |

| | | |
|---|---|---|
| | Tche | 1.000 |
| | CD4 count | 0.734 |
| **Fibrosis risk factors** | Age | 0.373 |
| | Male gender | 0.707 |
| | BMI | 0.210 |
| | Waist circumference | 0.902 |
| | Diabetes | 0.452 |
| | ALT | 0.210 |
| | AST | 0.210 |
| | Years of HIV infections | 0.902 |

**Table S4. Prevalence of NAFLD in PLWH in Europe by population**

| Overall | Sample size | Pooled Prevalence (95%CI) | p-value |
|---|---|---|---|
| **Overall analysis** | 10118 | 43% (37%-49%) | 0.000 |
| **Adult** | 10095 | 44% (38%-50%) | 0.000 |
| **Adolescent** | 23 | 17% (2%-33%) | 0.028 |

## Table S5. Effect size of Risk Factors

| | Variables | Sample size | p-value | SMD/OR(95%CI) |
|---|---|---|---|---|
| **NAFLD risk factors** | Age | 7444 | 0.253 | 0.30(-0.21,0.81) |
| | Gender | 7152 | 0.005 | 2.32(1.29,4.19) |
| | BMI | 7378 | 0.001 | 1.08(0.46,1.70) |
| | Waist circumference | 1852 | 0.000 | 1.54(0.95,2.14) |
| | Diabetes | 7329 | 0.000 | 3.19(2.02,5.05) |
| | Hypertension | 6181 | 0.000 | 1.95(1.65,2.32) |
| | Metabolic syndrome | 1447 | 0.000 | 3.19(2.39,4.26) |
| | Dyslipidemia | 5999 | 0.000 | 2.40(2.11,2.72) |
| | Triglyceride | 2727 | 0.000 | 0.58(0.27,0.89) |
| | HDL | 2016 | 0.005 | -0.44(-0.74,-0.13) |
| | LDL | 2067 | 0.008 | 0.13(0.03,0.23) |
| | ALT | 7275 | 0.000 | 0.44(0.21,0.67) |
| | AST | 6725 | 0.019 | 0.19(0.03,0.36) |
| | CD4 count | 6719 | 0.052 | 0.20(-0.00,0.39) |
| | CD4 nadir | 5675 | 0.733 | -0.03(-0.21,0.15) |
| | Years of HIV infection | 7444 | 0.155 | 0.22(-0.09,0.53) |
| | Years on ART | 6239 | 0.017 | 0.64(0.11,1.16) |
| | Undetectable HIV viral [illegible] | 4999 | 0.125 | 1.38(0.91,2.09) |
| **NASH risk factors** | Age | 293 | 0.765 | 0.05(-0.28,0.38) |
| | BMI | 293 | 0.000 | 0.68(0.35,1.02) |
| | ALT | 293 | 0.283 | 1.09(-0.90,3.09) |
| | AST | 293 | 0.336 | 1.13(-1.17,3.43) |
| | TG | 293 | 0.008 | 0.45(0.12,0.78) |
| | Tche | 293 | 0.085 | -0.29(-0.62,0.04) |
| **Fibrosis risk factors** | Age | 2736 | 0.003 | 0.35(0.12,0.58) |
| | Gender | 2685 | 0.049 | 1.32(1.00,1.73) |
| | BMI | 2580 | 0.000 | 0.74(0.44,1.04) |

| | | | |
|---|---|---|---|
| Waist circumference | 1308 | 0.000 | 0.56(0.38,0.74) |
| Diabetes | 2736 | 0.000 | 3.72(1.90,7.27) |
| ALT | 2029 | 0.032 | 0.66(0.06,1.27) |
| AST | 2580 | 0.001 | 0.75(0.32,1.18) |
| Years of HIV infection | 757 | 0.584 | 0.13(-0.33,0.59) |

Note: NAFLD: Nonalcoholic fatty liver disease; NASH: Nonalcoholic steatohepatitis; BMI: Body mass index; HDL: High density lipoprotein; LDL: Low density lipoprotein; ALT: Alanine transaminase; AST: Aspartate transaminase; TG: Triglyceride; Tche: Total cholesterol; ART: Anti-retroviral therapy

## 2 Supplementary Figures

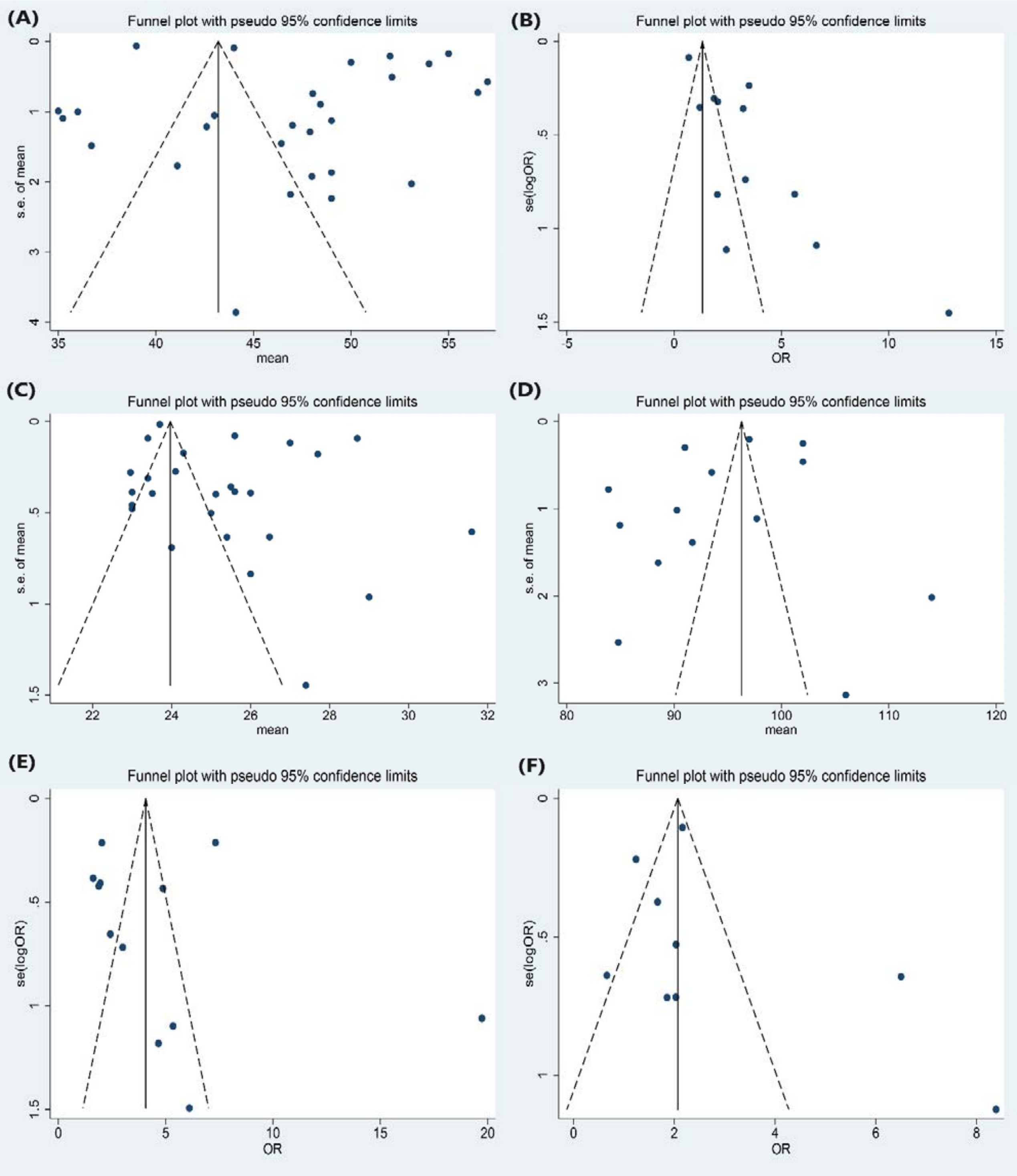


**Figure S1** Funnel plot for NAFLD risk factors. (A) Age; (B) Male gender; (C) BMI; (D) Waist circumference; (E) Diabetes; (F) Hypertension

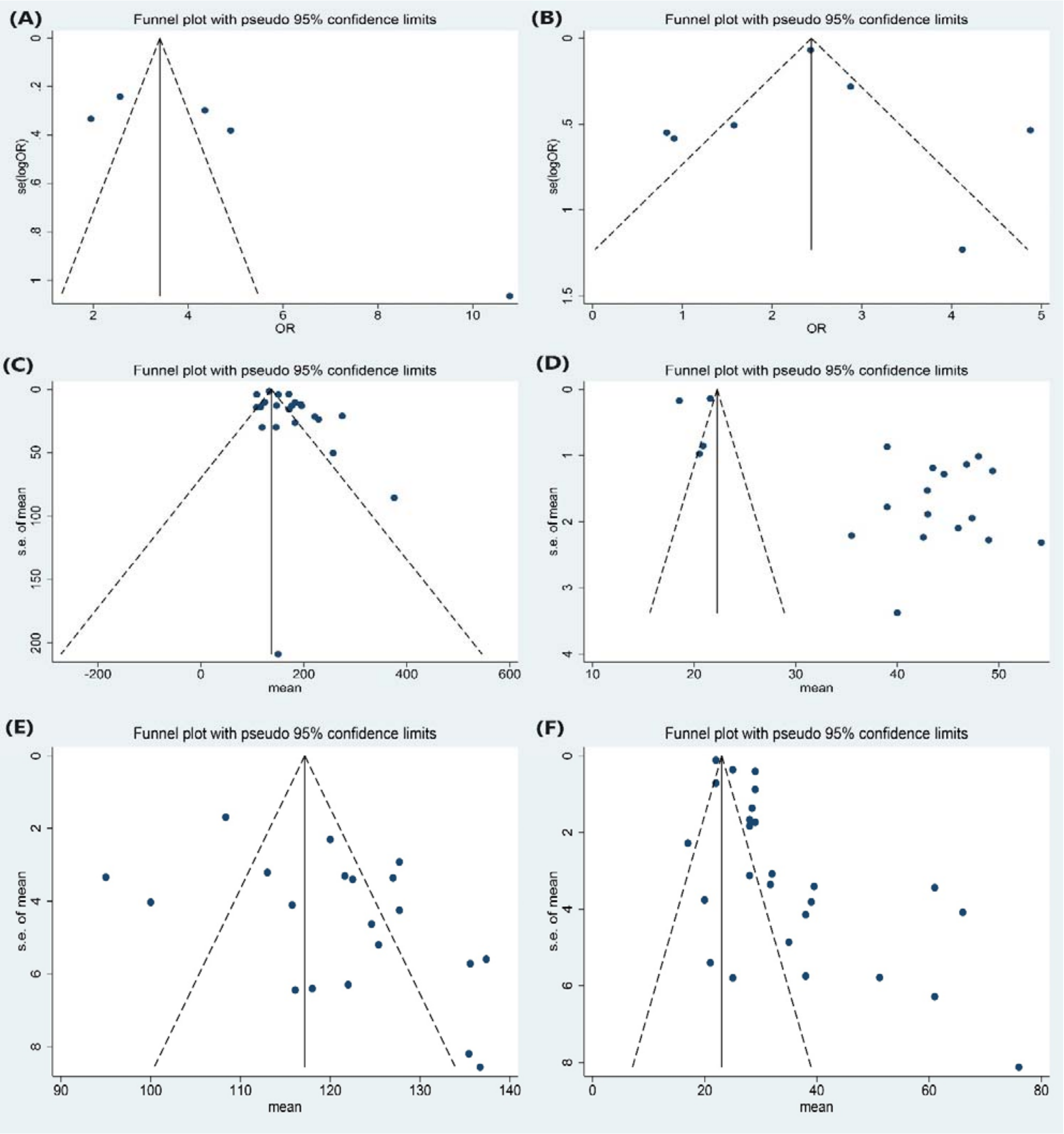


**Figure S2** Funnel plot for NAFLD risk factors. (A) Metabolic syndrome; (B) Dyslipidemia; (C) Triglycerides; (D) HDL; (E) LDL; (F) ALT;

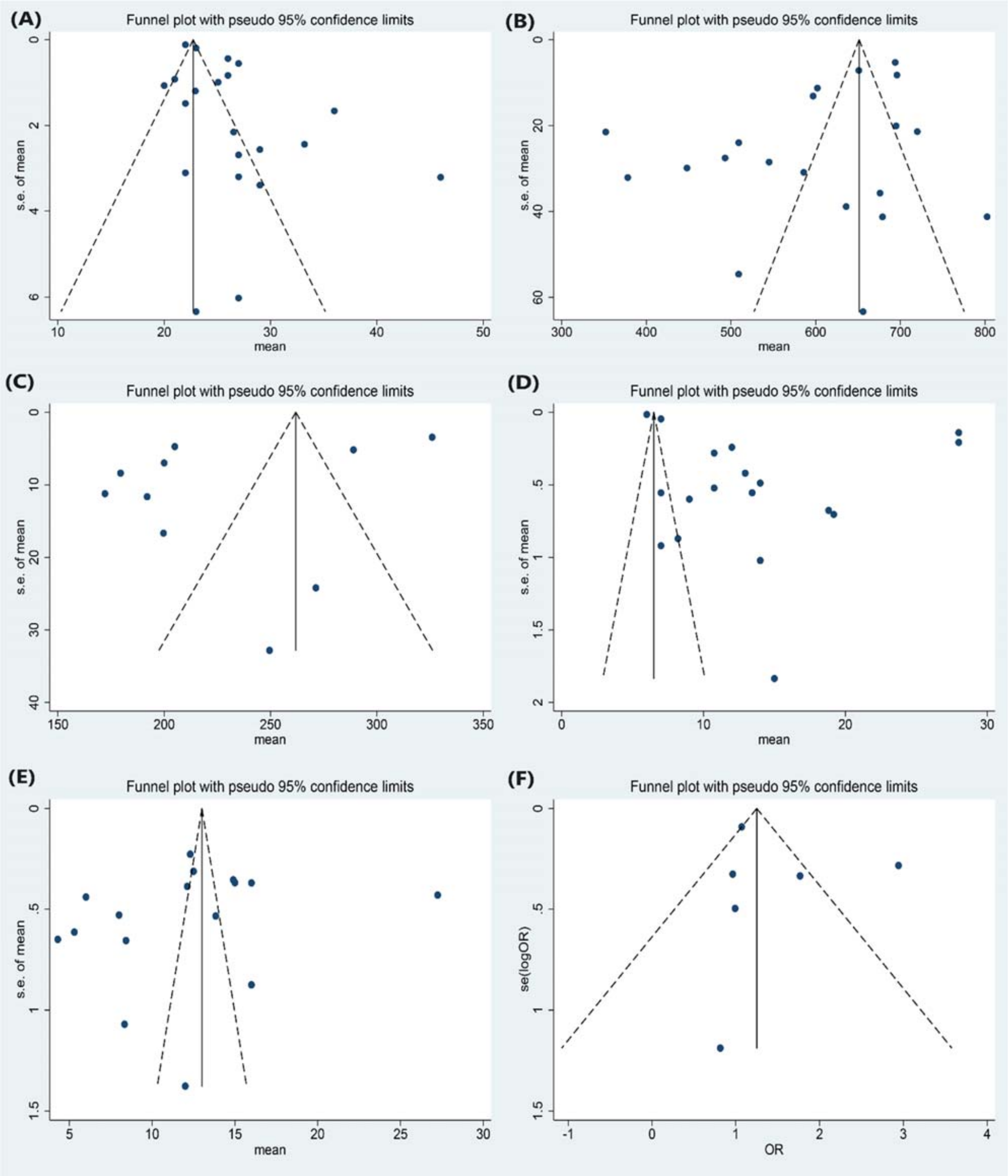


**Figure S3** Funnel plot for NAFLD risk factors. (A) AST; (B) CD4 count; (C) CD4 nadir; (D) Years of HIV infection; (E) Years on antiretroviral therapy; (F) Undetectable HIV viral load

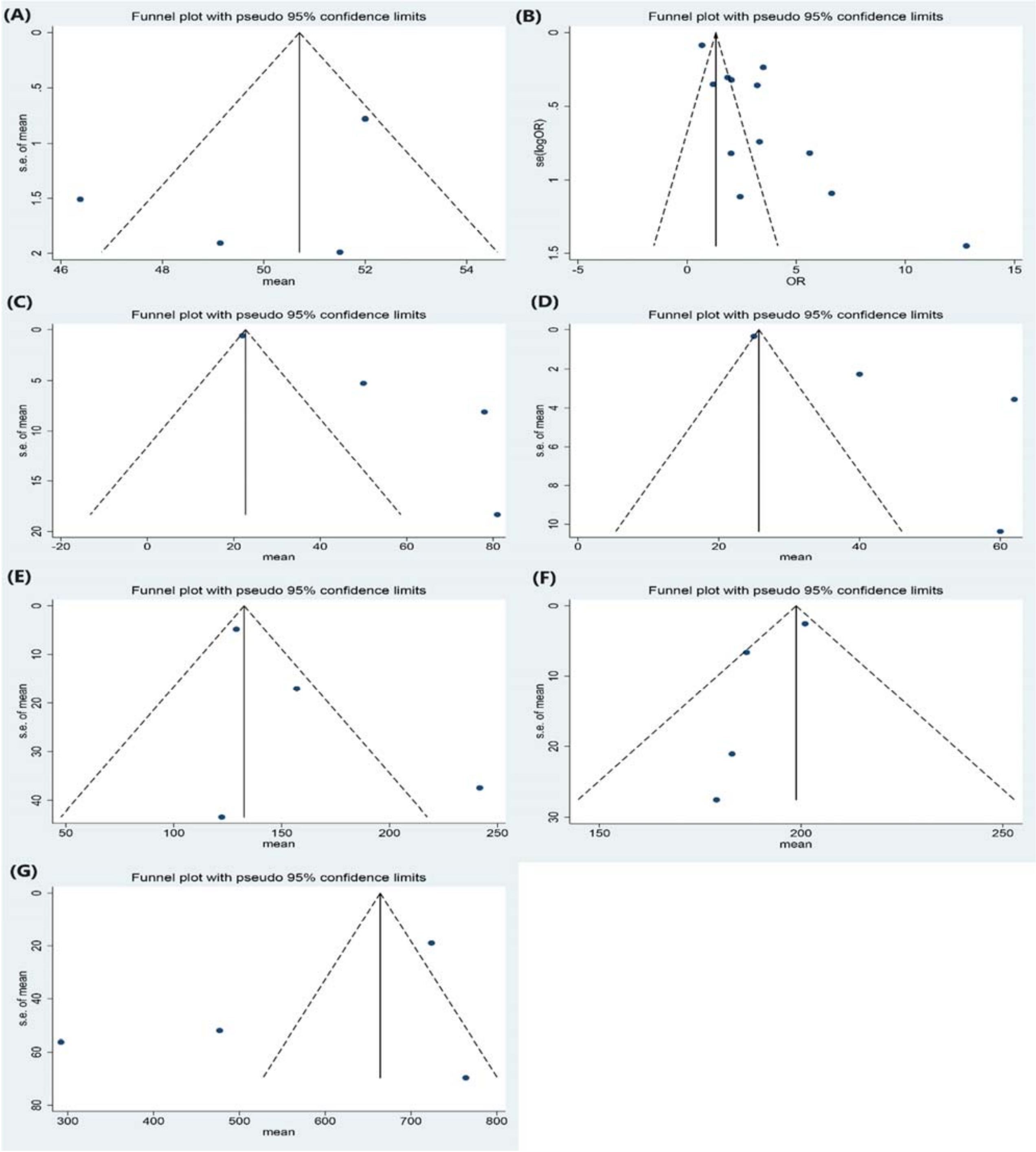


**Figure S4** Funnel plot for NASH risk factors: (A) Age; (B) BMI; (C) ALT; (D) AST; (E) Triglycerides; (F) Total cholesterol; (G) CD4 count

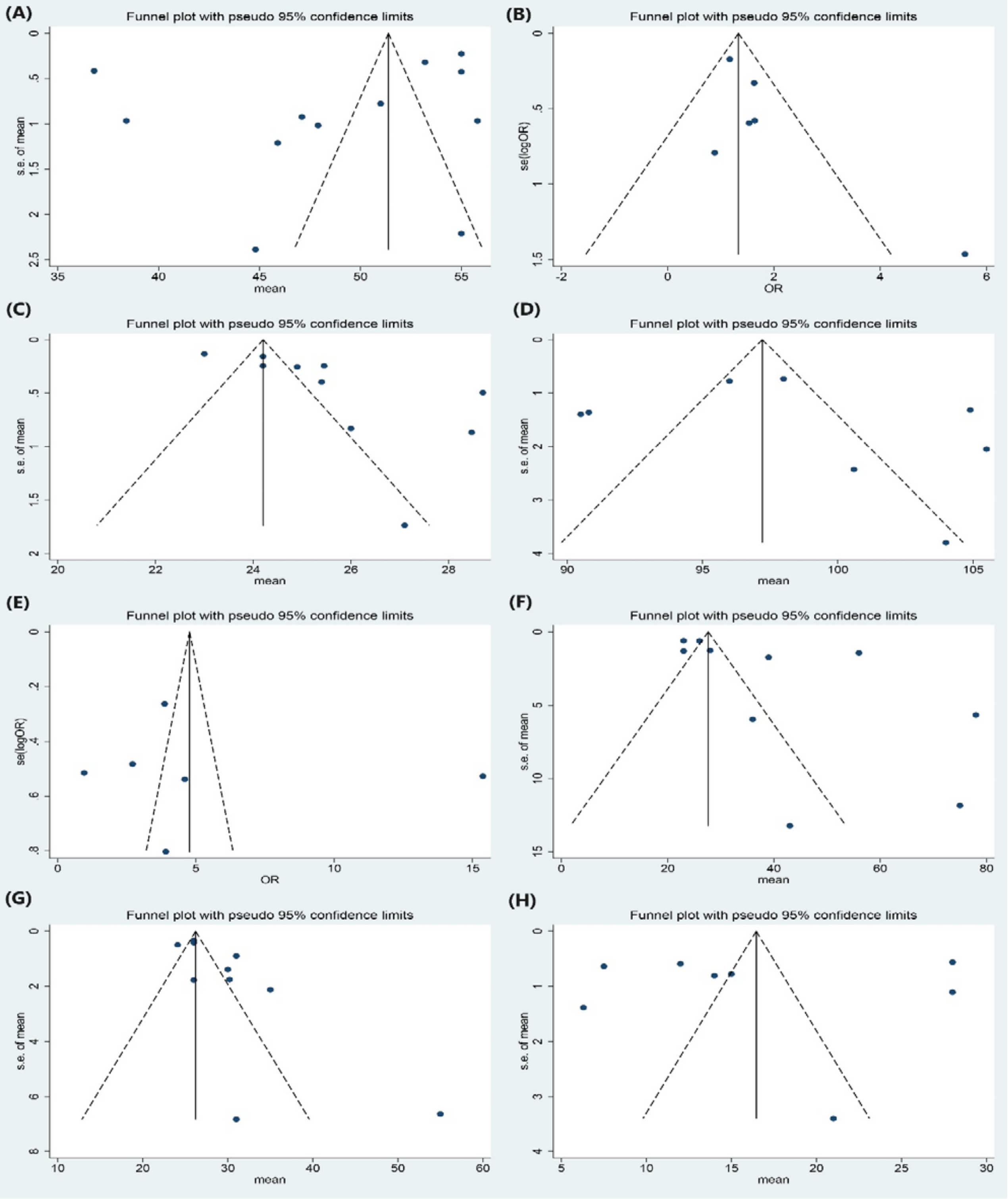


**Figure S5** Funnel plot for fibrosis risk factors: (A) Age; (B) Male gender; (C) BMI; (D) Waist circumference; (E) Diabetes; (F) ALT; (G) AST; (H) Years of HIV infection

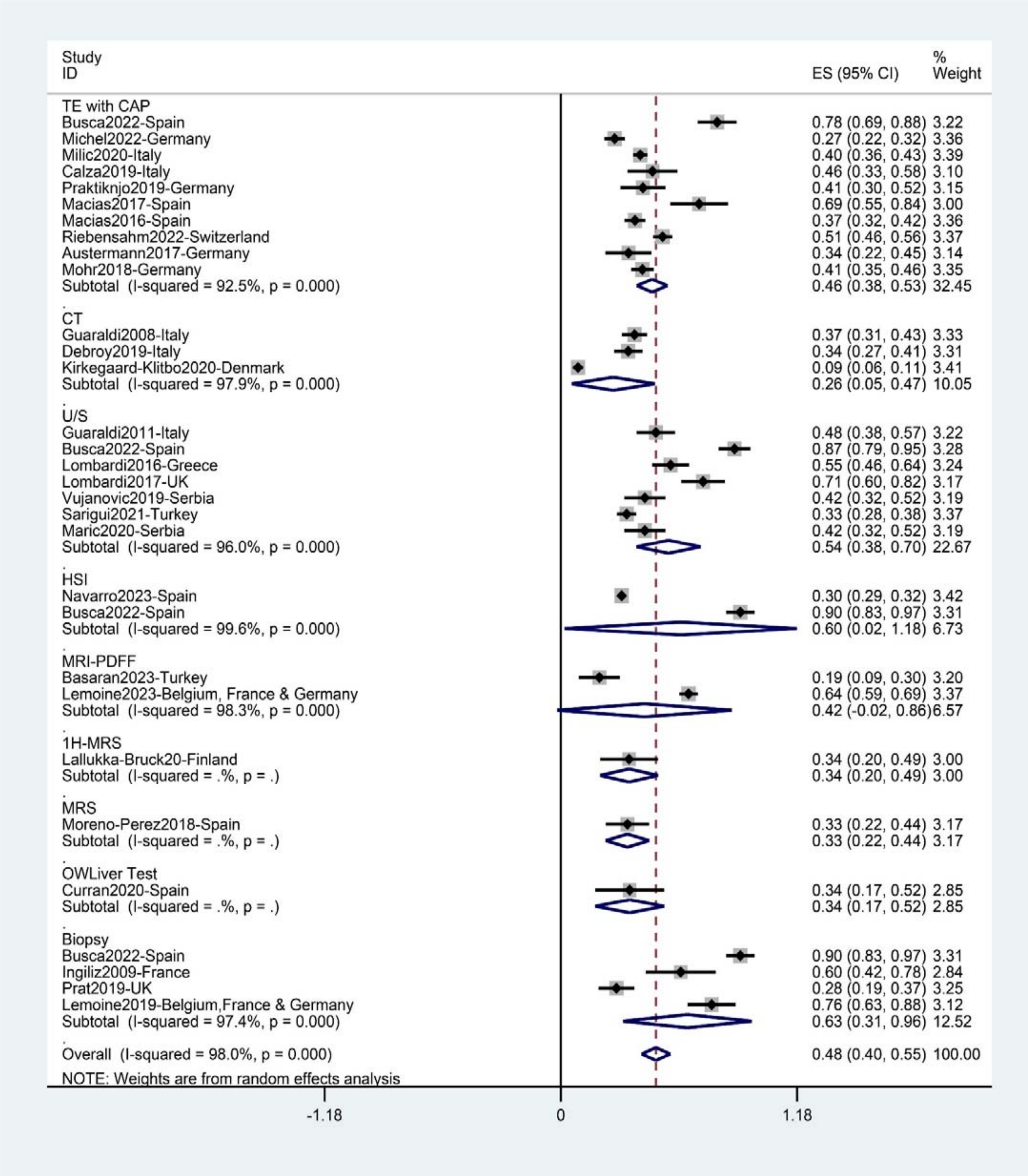


**Figure S6 Forest plot for NAFLD prevalence in PLWH in Europe subgrouped by diagnostic methods**

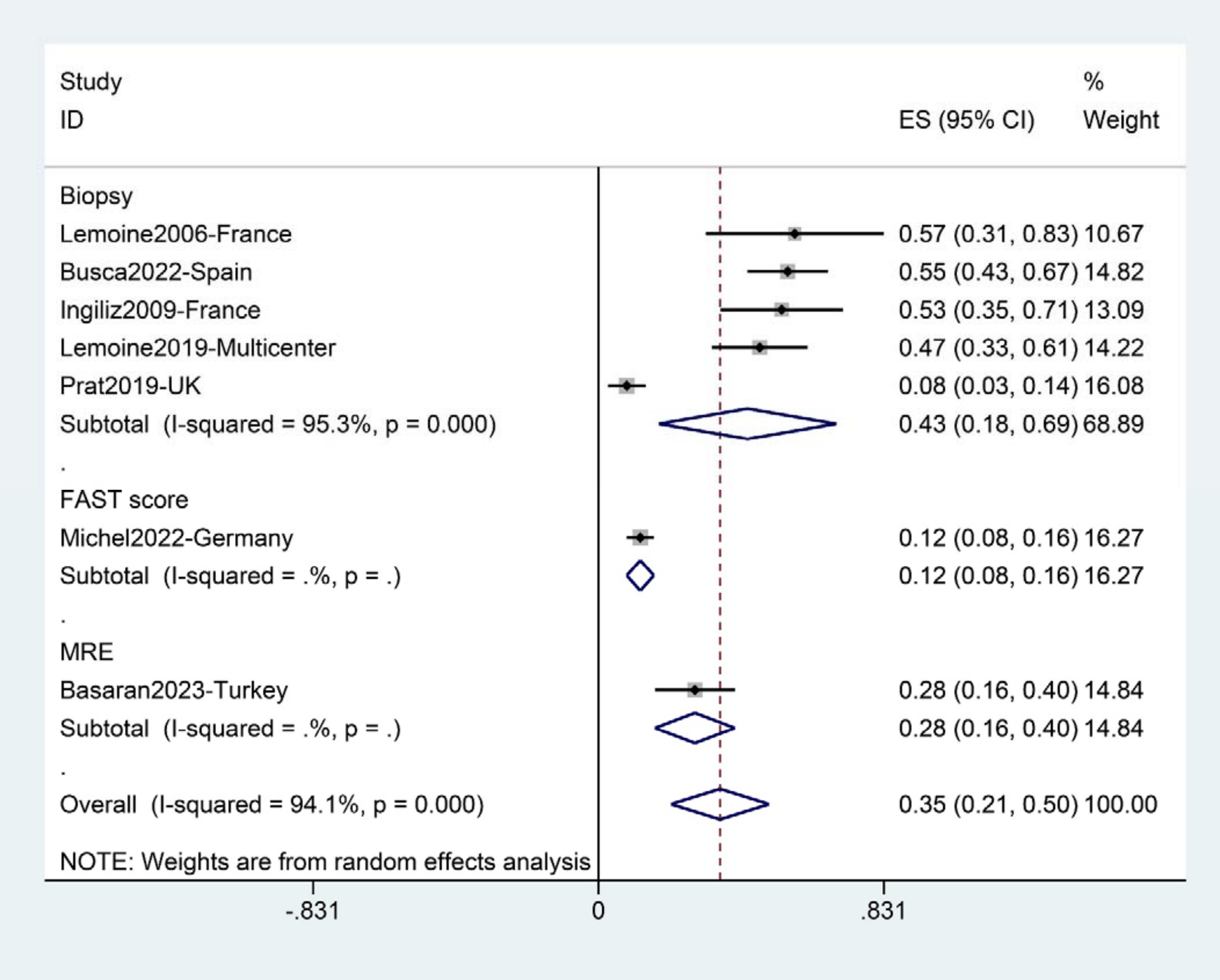


**Figure S7 Forest plot for NASH prevalence in PLWH in Europe subgrouped by diagnostic methods**

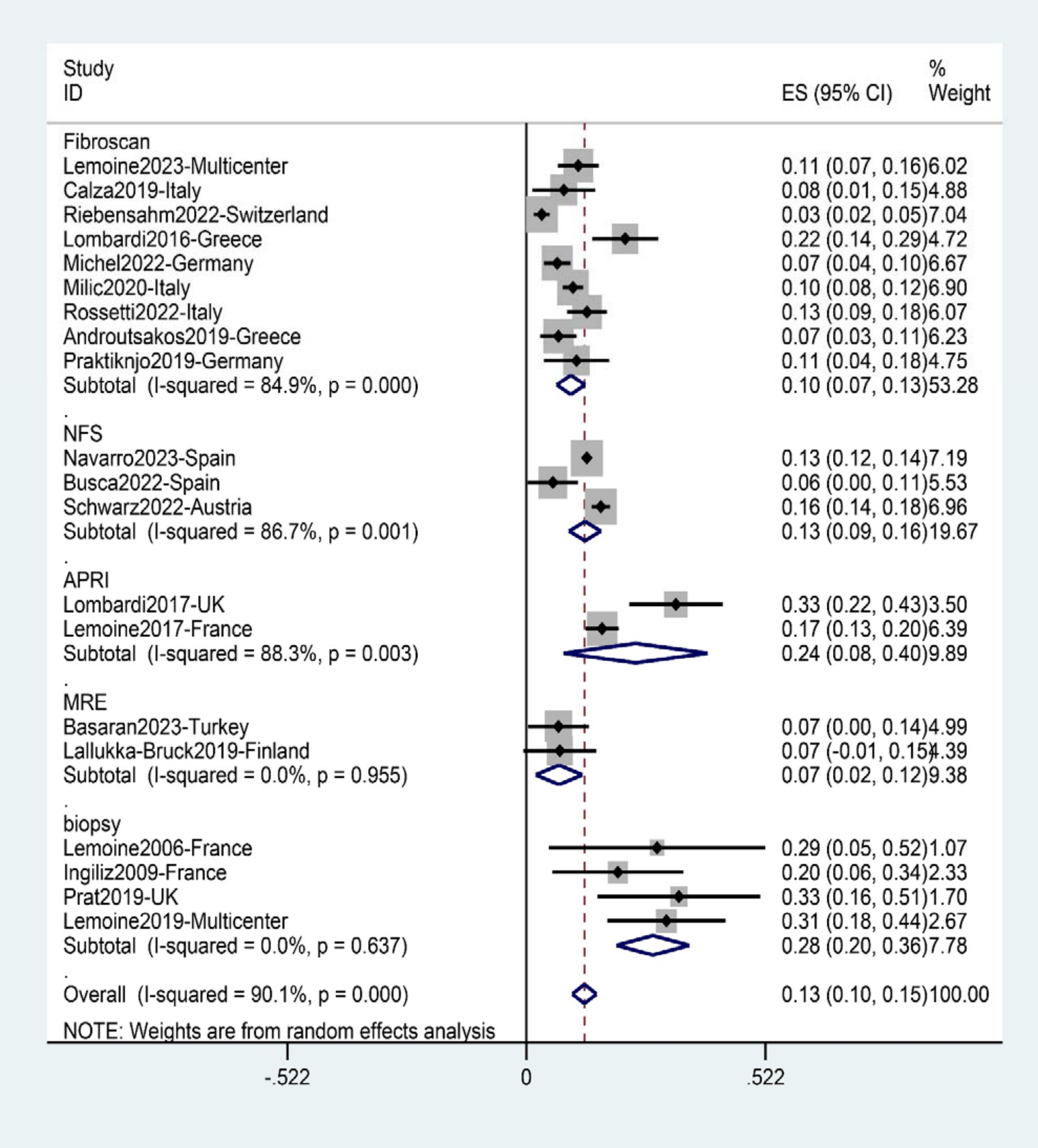


**Figure S8 Forest plot for liver fibrosis prevalence in PLWH in Europe subgrouped by diagnostic methods**
